# *Gossypium barbadense* genome sequence provides insight into the evolution of extra-long staple fiber and specialized metabolites

**DOI:** 10.1038/srep14139

**Published:** 2015-09-30

**Authors:** Xia Liu, Bo Zhao, Hua-Jun Zheng, Yan Hu, Gang Lu, Chang-Qing Yang, Jie-Dan Chen, Jun-Jian Chen, Dian-Yang Chen, Liang Zhang, Yan Zhou, Ling-Jian Wang, Wang-Zhen Guo, Yu-Lin Bai, Ju-Xin Ruan, Xiao-Xia Shangguan, Ying-Bo Mao, Chun-Min Shan, Jian-Ping Jiang, Yong-Qiang Zhu, Lei Jin, Hui Kang, Shu-Ting Chen, Xu-Lin He, Rui Wang, Yue-Zhu Wang, Jie Chen, Li-Jun Wang, Shu-Ting Yu, Bi-Yun Wang, Jia Wei, Si-Chao Song, Xin-Yan Lu, Zheng-Chao Gao, Wen-Yi Gu, Xiao Deng, Dan Ma, Sen Wang, Wen-Hua Liang, Lei Fang, Cai-Ping Cai, Xie-Fei Zhu, Bao-Liang Zhou, Z. Jeffrey Chen, Shu-Hua Xu, Yu-Gao Zhang, Sheng-Yue Wang, Tian-Zhen Zhang, Guo-Ping Zhao, Xiao-Ya Chen

**Affiliations:** 1 Esquel Group, 25/F Eastern Cenrtal Plaza, 3 Yin Hing Road, Shau Kei Wan, Hongkong, China; 2 National Key Laboratory of Plant Molecular Genetics, National Plant Gene Research Center, Institute of Plant Physiology and Ecology, Shanghai Institutes for Biological Sciences, Chinese Academy of Sciences, Shanghai 200032, China; 3 Shanghai–Ministry of Science and Technology Key Laboratory of Health and Disease Genomics, Chinese National Human Genome Center at Shanghai, Shanghai 201203, China; 4 Nanjing Agricultural University, Nanjing, Jiangsu 210095, China; 5 State Key Laboratory of Genetic Engineering, School of Life Sciences, Fudan University, Shanghai 200433, China; 6 The Institutes of Biology and Medical Sciences, Soochow University, Suzhou, Jiangsu 214123, China; 7 Max Planck Independent Research Group on Population Genomics, Chinese Academy of Sciences and Max Planck Society (CAS-MPG) Partner Institute for Computational Biology (PICB), Shanghai Institutes for Biological Sciences, Chinese Academy of Sciences, Shanghai 200031, China; 8 Institute for Cellular and Molecular Biology and Center for Computational Biology and Bioinformatics, The University of Texas at Austin, Austin, Texas 78712, USA

## Abstract

Of the two cultivated species of allopolyploid cotton, *Gossypium barbadense* produces extra-long fibers for the production of superior textiles. We sequenced its genome (AD)_2_ and performed a comparative analysis. We identified three bursts of retrotransposons from 20 million years ago (Mya) and a genome-wide uneven pseudogenization peak at 11–20 Mya, which likely contributed to genomic divergences. Among the 2,483 genes preferentially expressed in fiber, a cell elongation regulator, *PRE1*, is strikingly A_t_ biased and fiber specific, echoing the A-genome origin of spinnable fiber. The expansion of the PRE members implies a genetic factor that underlies fiber elongation. Mature cotton fiber consists of nearly pure cellulose. *G. barbadense* and *G. hirsutum* contain 29 and 30 cellulose synthase (CesA) genes, respectively; whereas most of these genes (>25) are expressed in fiber, genes for secondary cell wall biosynthesis exhibited a delayed and higher degree of up-regulation in *G. barbadense* compared with *G. hirsutum*, conferring an extended elongation stage and highly active secondary wall deposition during extra-long fiber development. The rapid diversification of sesquiterpene synthase genes in the gossypol pathway exemplifies the chemical diversity of lineage-specific secondary metabolites. The *G. barbadense* genome advances our understanding of allopolyploidy, which will help improve cotton fiber quality.

Whole-genome duplication (WGD) or polyploidy is a primary driving force in the evolution of many eukaryotic organisms, especially flowering plants^1^,^2^,^3^,^4^. Many crops are neo-allopolyploids that harbor different sets of genomes^5^,^6^, including the cultivated Upland cotton *Gossypium hirsutum* (AD)_1_ and the extra-long staple (ELS) cotton *Gossypium barbadense* (AD)_2_. However, our understanding of the molecular mechanism that facilitates the success of allopolyploids and the formation of agronomic traits remains limited.

Cotton provides the most important raw material for the textile industry and consequently profoundly affects the world economy and daily human life. The cotton genus *Gossypium* contains 45 diploid (2n = 26) and six tetraploid (2n = 52) species^7^,^8^, among which only four species, including two tetraploids (*G. hirsutum* and *G. barbadense*) and two diploids (*G. herbaceum* and *G. arboreum*), produce spinnable fiber. Diploid cottons are divided into eight cytogenetic genome groups, A-G and K. The sizes of genomes vary between groups due to the lineage-specific proliferation of retrotransposons^7^. The D-group species have the smallest genome with *G. raimondii* (D_5_) of less than 880 Mb^9^,^10^,^11^, whereas the genome of *G. arboreum* (A_2_) in the A-group is approximately 1,700 Mb^12^. *G. hirsutum* and *G. barbadense* are considered classic natural allotetraploids that originated in the New World approximately 2 million years ago (Mya) from trans-oceanic hybridization between an A-genome ancestral African species, *G. herbaceum* (A_1_) or *G. arboreum* (A_2_), and a native D-genome species, *G. raimondii* or *G. gossypioides* (D_6_)^13^, followed by divergence from their common ancestor (Fig. 1). These two allotetraploids are likely the oldest major allopolyploid crops^10^,^14^,^15^.

Cotton fiber is derived from single-celled, seed-borne hair (trichrome), and the development of fiber cells is largely synchronized in a cotton ball (fruit) in four overlapping stages: initiation, elongation, secondary cell wall synthesis and maturation^16^. These processes provide an excellent model to dissect cell differentiation, elongation and cellulose biosynthesis. The rate and duration of the elongation stage determines fiber length, and the secondary cell wall biosynthesis affects fiber strength and fineness^17^,^18^. The Upland cotton *G. hirsutum* constitutes ~90% of the annual cotton output and is characterized by its high yield yet moderate fiber qualities, whereas the ELS cotton *G. barbadense* produces over 5% of the world’s cotton and is famous for its superior quality fiber, as based on the length, strength and fineness of its fibers (Fig. 1). Therefore, *G. barbadense* is preferred for the production of high-grade or special cotton textiles.

Although *G. barbadense* and *G. hirsutum* may share a common progenitor, the two species substantially differ, which has hindered the transfer of the superior fiber traits of *G. barbadense* to *G. hirsutum via* inter-species hybridization. This transfer has been particularly hindered by distorted segregation^19^. The recently released genome sequences of *G. hirsutum*^20^,^21^ and the two extant diploid progenitor species, *G. raimondii*^10^,^11^ and *G. arboreum*^12^, have provided insight into cotton evolution and a wealth of resources for fiber improvement. A genome sequence of *G. barbadense* will further our understanding of the dynamics of genome structures and the genetic driving force associated with allotetraploids, particularly the molecular basis of the formation of fibers with superior traits.

## Results

### Genome sequence and assembly

We adopted a progressive strategy to sequence the allotetraploid genome of *G. barbadense* cv. Xinhai21 (AD)_2_. First, the genomes of the extant diploid species of *G. arboreum* (A_2_) and *G. raimondii* (D_5_) were separately sequenced and assembled. These sequences, together with their published genomes^10^,^12^, were used as references for early assortments of the primary reads into A_t_ and D_t_ subgenomes. Then the sequences were assembled into A_t_ and D_t_ contigs and scaffolds (Supplementary Table 1). A total of 471 Gb (188× genome equivalent) of data were separately produced using the Roche 454, Illumina Hiseq2000 and PacBio SMRT sequencing platforms (Supplementary Table 2). The particularly long reads (22.67 Gb) obtained from PacBio SMRT and the assembled 53-Gb contigs of the BAC pool further reduced the effects of repeats in the assembly, yielding a gap reduction of 63.4% (Supplementary Fig. 1). Finally, we used the ultra-dense linkage map consisting of 4,999,048 single-nucleotide polymorphism (SNP) loci^22^ to assign and orient the 26 chromosomes and validate the polyploidy genome of *G. barbadense* (Supplementary Fig. 2). We detected only 20 Mb sequences in which the subgenome classification of homoeologous sequences conflicted between the sequence assembly and the linkage mapping strategies, which was likely due to sequence conversions between the two subgenomes. A total of 208 Mb sequences with erroneous inter-chromosomal joins in the A_t_ or D_t_ subgenome were detected and then corrected.

The combination of these methods resulted in a draft genome for *G. barbadense* with an overall contig N50 of 72 kilobases (kb) and scaffold N50 of 503 kb covering 1.395 Gigabases (Gb) of the A subgenome (A_t_) and 0.776 Gb of the D subgenome (D_t_) (Table 1 and Fig. 2). In total, ~88% of the 2.470 Gb genome was based on k-mer estimation (Supplementary Fig. 3). The genome contains at least 63.2% repeated sequences (Supplementary Table 3), half of which are transposable elements (TEs) that primarily consist of long-terminal-repeat retrotransposons (LTR retrons) (Supplementary Fig. 4).

### Gene annotation

To initiate gene prediction, ~1 million expressed sequence tags (ESTs) that were generated using Roche 454 from a combination of 28 samples of eight tissues/organs collected at different development stages were mapped to the genome as gene models, which resulted in 40,502 and 37,024 protein-coding genes (CDSs) with an average length of 1,077 and 1,123 bp in the *G. barbadense* A_t_ and D_t_ subgenomes, respectively (Table 1), and falling in the same range as the number and length of CDSs of *G. raimondii*^10^,^11^. Further evaluation using the 70-Gb RNA-Seq data *via* Illumina supported 96.6% of the predicted CDSs. The 77,526 predicted genes were annotated, which revealed 62,966 functional genes, excluding 8,518 A_t_ and 6,042 D_t_ genes (~20%) that lacked clear biological functions.

To examine the influence of allopolyploidy on gene contents, we classified cotton genes into domain families. The composition and family size of the assigned Pfam domain families are overall identical in *G. barbadense* A_t_ and D_t_, *G. raimondii* and, to a lesser extent, *G. arboreum*. Protein domains whose function was clearly annotated, such as protein kinase, cytochrome P450, and pentatrico-peptide repeat (PPR), were commonly over-represented as large families (Supplementary Table 4 and Supplementary Fig. 5) as in other angiosperm plants^23^,^24^,^25^. Although most domains (3,039 out of 3,674) were maintained in each subgenome after the two were merged, pronounced changes in family size occurred, as exemplified by more ring finger domain (PF13639) and leucine rich repeat (PF13855) genes in the diploid D genome than in either A_t_ or D_t_ (Supplementary Table 4). This finding suggested that super-large families have evolved faster than others and tended to lose members in polyploids^26^.

### Genome evolution

A total of 21,639 pairs of orthologs were identified between A_t_ and D_t_. We compared the *Ks* values of orthologous gene pairs among *G. barbadense* (Gb), *G. hirsutum* (Gh) and *G. raimondii* (Gr) at the whole-genome level (Fig. 3a and Supplementary Table 5). A peak of 0.011 in both GbD_t_:GrD_5_ and GhD_t_:GrD_5_ indicates that the D_t_ subgenome in of both allotetraploids originated from a *G. raimondii*-like progenitor^27^. The peak values for GbA_t_:GaA_2_ and GhA_t_:GaA_2_ are lower but again similar, presumably due to a shorter time since divergence compared to that between D-genome species. In addition, unlike *G. raimondii*, which is a wild species, *G. arboreum* has long been cultivated in African and Asian countries. Another pair of similar *Ks* peaks (0.005) of GbA_t_:GhA_t_ and GbD_t_:GhD_t_ further supports the common origin of the two allotetraploid cottons and suggests their later divergence approximately 1 Mya (Fig. 3a). Based on the larger *Ks* value (0.04) for A_t_:D_t_, we estimated the divergence time between the *Gossypium* A- and D-genome species to be approximately 8 Mya, consistent with previous estimates that were based on a few single-copy genes^13^,^27^. The *Ks* values of paralogs in the two subgenomes of *G. barbadense* both peak at 0.4–0.5, which indicate ancient WGD event(s) that occurred 50–70 Mya (Fig. 3b), which were responsible for the repeated genome expansion in *Gossypium* after divergence from the *Theobroma cacao* lineage more than 60 Mya^10^.

Both the A_t_ and D_t_ subgenomes of *G. barbadense* demonstrate a high level of co-linearity with the *G. raimondii* genome^10^,^11^ (Supplementary Fig. 6). A total of 21 Megabase (Mb) sequences in the D_t_ and 7.4 Mb in the A_t_ were identified as inter-subgenome translocation regions (Supplementary Fig. 7). Two of three major intra-subgenomic rearrangements between chrA2/chrA3 and chrA4/chrA5^28^,^29^ were observed in the A_t_ of both of the allotetraploid cottons but absent in the D_t_ or *G. raimondii* genome (Fig. 2), suggesting that the two translocations likely occurred after the separation of the A and D genomes.

### Genomic plasticity and evolution

We identified 6,014/2,422 complete LTR retrons with an average length of 9,256/8,130 bp in A_t_/D_t_ (Supplementary Tables 6 and 7), similar to the numbers of LTR retrons in *G. hirsutum* A_t_ and D_t_, *G. arboreum* and *G. raimondii* (Supplementary Table 8). The singleton LTR retrons ratio is 83.5% in A_t_ and 82.2% in D_t_ (compared with 85.4% in *G. raimondii* and 73.2% in *G. arboreum*), close to that (86%) in the genome of a gymnosperm tree, *Picea abies*^30^ (an indication of high divergence).

**Figure 1:**
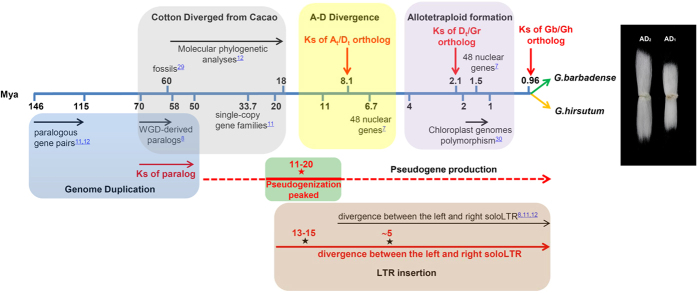
A schematic map of the evolution of allotetraploid cottons. Allotetraploid cotton evolved from the natural hybridization between A- and D-genome species and has split into six species, including the widely cultivated *G. barbadense* (AD_2_) and *G. hirsutum* (AD_1_). Evolutionary time (in Mya) is indicated by a numbered axis; major evolutionary events are represented by arrows and concluded in boxes. A black star indicates a retrotransposon burst, and a red star indicates a boom in pseudogene production. Gr, *G. raimondii*, a diploid species (D_5_); Gb, *G. barbadense*; Gh, *G. hirsutum*. Mature cotton fiber is shown for extra-long stable (ELS) cotton (*G. barbadense*, AD_2_) and Upland cotton (*G. hirsutum*, AD_1_).

The TE proliferations in *G. barbadense* and *G. hirsutum*^20^,^21^, represented by insertions of LTR retrons based on estimations according to the sequence divergence between the left and right soloLTR^31^, have increased since 20 Mya, and three distinct bursts were identified. Interestingly, the first two bursts appear to successively pre-date the divergence and the re-unification of the diploid A/D genomes (Fig. 3c). The LTR retrons clearly show type-specific and subgenome-biased proliferations (Fig. 3c). Their insertion rates in the A genome appear consistently higher than those in the D genome. For example, a large number (9.15%) of LTR retrons burst at 5 Mya and decreased thereafter in A_t_, whereas a substantially lower and flat peak appeared 3–5 Mya in D_t_ (Fig. 3c). This peak at least partly accounts for the 1.7-fold more LTR retrons in the former genome. However, the faster loss of LTR retrons in the D genome may also be responsible for genome size variations and the different rates of genome expansion^32^. Notably, the third asymmetric activities of transposons differ between *G. barbadense* and *G. hirsutum* (Fig. 3c), which suggests a possible cause of subgenome divergence that may have promoted the speciation of allotetraploid cottons beginning approximately 1 Mya (Fig. 1). These observations indicate that the genome-specific differential dynamics of TE proliferations could be a major force that has driven the rapid evolution and diversification of *Gossypium* species, which may also be inferred in other flowering plants.

### Pseudogenization prior to and after polyploidization

Pseudogenes are disabled copies resembling functional genes that have been retained in the genome^26^,^33^. They can be grouped into three categories: duplicated (derived from gene duplication), processed (generated by the integration of reversed-transcribed cDNAs into genomes) and fragmented (neither processed nor duplicated)^33^. To further investigate the influence of TE bursts and polyploidization on the cotton genomic architecture, we predicted pseudogenes in *G. barbadense* (Supplementary Table 9) and classified them into the three categories (Supplementary Fig. 8), most of which are silenced without any detectable transcripts in all tissues examined.

Each subgenome of *G. barbadense* contains more predicted pseudogenes than the diploid genome of *G. raimondii* (Supplementary Table 9 and Supplementary Fig. 8), implying an accelerated pseudogenization after allopolyploid formation. A substantial portion of the pseudogenes in A_t_ and D_t_ showed a high sequence identity (above 90%, for example) with their parental genes (Supplementary Fig. 9), suggesting an insufficient duration for degeneration in recently formed pseudogenes. As expected, the *Ka/Ks* distributions indicate a substantially weaker natural selection on pseudogenes than on protein-coding genes (Supplementary Fig. 10), which is likely due to a loss of function in pseudogenes. The *Ks* value peaks at 0.06–0.1 corresponding to 11–20 Mya (Fig. 3d) and this boom of pseudogenization correlates with an LTR retron burst prior to the divergence of the A and D genomes (Fig. 3c). The average expression levels of the genes with LTR retron insertion within a 20-kb region upstream of the start codon are generally lower (RPKM = 7.72) than those of genes lacking this insertion (RPKM = 13) (Supplementary Table 10). Therefore, LTR retrons negatively affect the expression of nearby genes, which may promote pseudogenization. These results suggest that cotton progenitors likely lost genes and experienced LTR retron bursts following the ancient WGD, which promoted diversification in *Gossypium* genomes; however, the role of TE-associated pseudogenization in the stabilization of subgenomes in polyploids requires a more detailed analysis.

### Extra-long staple fiber formation

We identified 2,483 and 1,879 genes that are specifically or preferentially expressed in fibers and the ovule, respectively (Supplementary Tables 11 and 12). The highly active genes in the ovule are abundant in the protein families of nucleic acid binding/transcription factor activity and nutrient reservoir activity, whereas the up-regulated genes of fibers are enriched in several categories, such as those related to cytoskeleton, carbohydrate metabolism, cell wall biosynthesis and cellulose biosynthesis function (Supplementary Tables 13 and 14).

**Figure 2:**
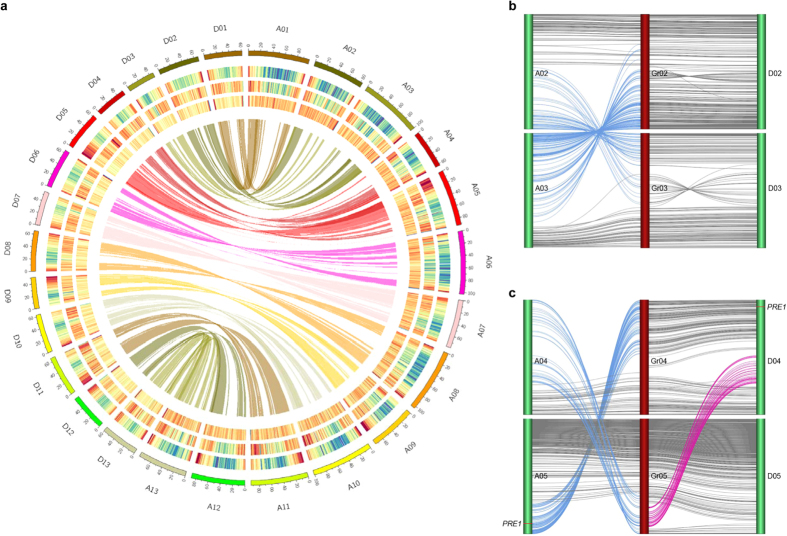
*G. barbadense* genome atlas and chromosome-level translocations. (**a**) Genome atlas. The outermost circle represents the numbered chromosomes of A_t_ and D_t_, and chromosome sizes are marked by a scale plate. The three tracks moving inside successively represent gene, peudogene and repeat densities (calculated with 1 Mb windows) across the chromosomes. The core ribbon-link shows collinearity between A_t_ and D_t_. (**b**,**c**) chromosomal translocations. The translocations among chromosome 2 and chromosome 3 of either A_t_ or D_t_ are indicated with blue lines (**b**) and those among chromosome 4 and chromosome 5 with blue and purple lines (**c**). The vertical colored lines from left to right represent chromosomes. The loci of *PRE1* implicated in fiber cell elongation are specifically marked with red in the chromosomes A05 and D04. Digits (01 to 13) after A, D or Gr indicate the chromosome of the A_t_/D_t_ subgenome of *G. barbadense* or of *G. raimondii*, respectively.

**Figure 3:**
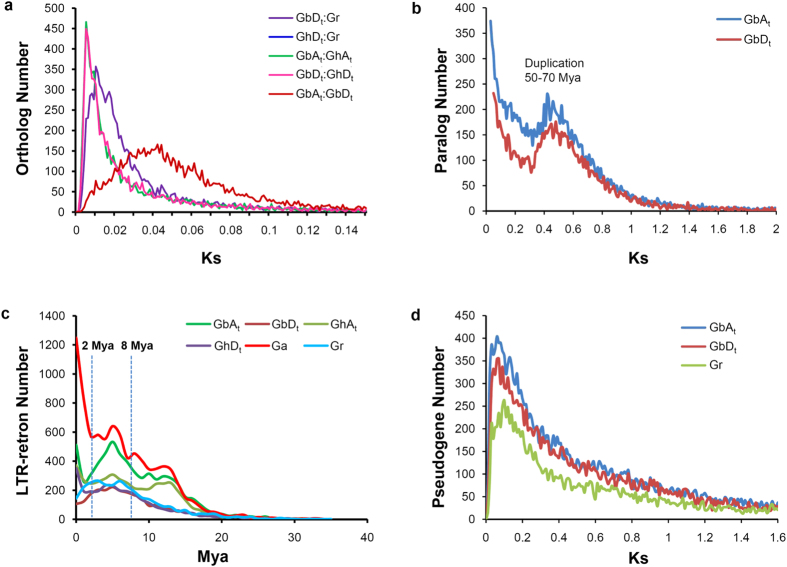
Evolutionary analysis of the *G. barbadense* genome. (**a**) *Ks* distribution of orthologs in cotton genomes. Data are grouped into 0.001 Ks units. (**b**) *Ks* distribution of paralogs in the *G. barbadense* genome. Data are grouped into 0.01 Ks units, and the peak region corresponds to 50–70 million years. (**c**) The distribution curve of the insertion times in the LTR retrons in the *G. barbadense* genome. The LTR retrons bursts are separated by dashed lines. (**d**) *Ks* distribution of pseudogenes with their closest functional paralogous genes. Data are grouped into 0.001-Ks units. The genomes of allotetraploid cottons are labeled using A_t_/D_t_, and the genomes of *G. arboreum* (A_2_) and *G. raimondii* (D_5_) are labeled using Ga and Gr.

**Figure 4:**
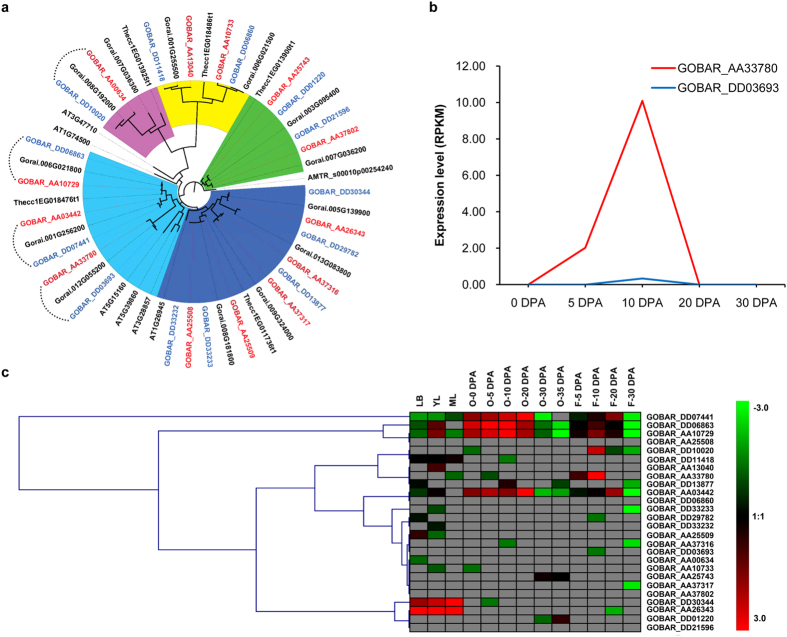
Expansion and diversification of *PRE* genes in *Gossypium*. (**a**) Phylogenetic analysis of *PRE* family genes in *Amborella trichopoda*, *Arabidopsis thaliana*, *G. raimondii* and *G. barbadense*. Subfamilies are overlaid with different colors, and the curved dotted lines indicate homoeologous gene pairs expressed in fiber. (**b**) *GbPRE1* (GOBAR_AA33780, GOBAR_DD03693) is a fiber-specific gene with strong A_t_ bias expression. The expression levels (RPKM) in ovules (0 DPA) and fiber cells (5, 10, 20, and 30 DPA) are shown. Detailed expression data are provided in Supplementary Table 10. (**c**) Hierarchical clustering analysis of expression of *PRE* genes in *G. barbadense*. LB, leaf bud; YL, young leaf; ML, mature leaf; O, ovule; F, fiber; DPA, days post-anthesis.

Consistent with a previous report^34^, equal numbers of genes in the A_t_ and D_t_ subgenomes demonstrated biased expression patterns (Supplementary Tables 15 and 16). Transcription factors play an important role in controlling agronomic novelty, and the MYB and homeodomain-containing factors have been shown to be key regulators of cotton fiber traits development^10^,^35^,^36^,^37^. We then analyzed transcription factor genes expressed in *G. barbadense* fiber in detail (Supplementary Table 17 and Supplementary Fig. 11). ***P***aclobutrazol ***Re***sistance (*PRE*) genes encode a group of transcription regulators known in other plants to promote cell elongation^38^,^39^,^40^. We identified 13 *PRE* family genes in *G. raimondii*; their 26 orthologous genes were recovered in *G. barbadense*. Analyzing the *PRE*-containing synteny blocks in plants revealed that cacao^41^ has five *PRE* genes, each of which has at least two orthologs in the *Gossypium* diploid genomes or the allotetraploid subgenomes (Fig. 4a and Supplementary Fig. 12). This expansion of *PRE* genes in cotton may have occurred during a complex 5–6-fold polyploidy process^10^,^11^, which was followed by differential gene loss but the retention of the ancient orthologs. Interestingly, two *PRE* genes are located in the two A_t_ translocation regions (chrA2/chrA3 and chrA4/chrA5) (Fig. 2c and Supplementary Fig. 12). In cotton, *PRE* genes are preferentially expressed in young tissues (Fig. 4b,c), which is consistent with their role in controlling cell size. Moreover, the expression of A_t_ and D_t_ *PRE* homoeologous genes was biased in *G. barbadense* (Supplementary Tables 11–12). In particular, the expression level of A_t_-subgenome *PRE1* was high and fiber specific, whereas the expression the D_t_ homoeolog was nearly undetectable (Fig. 4b). The A_t_-specific expression of a cell growth regulator provides a clue to support the origin or early evolution of spinnable fiber in the A-genome species^10^,^11^. The expansion and subsequent selection^11^,^34^ of *PRE* genes in *Gossypium* may have increased their regulatory activity and recruited specific member(s) for the rapid and extensive elongation of cotton fiber (Figs 1 and 4c).

**Figure 5:**
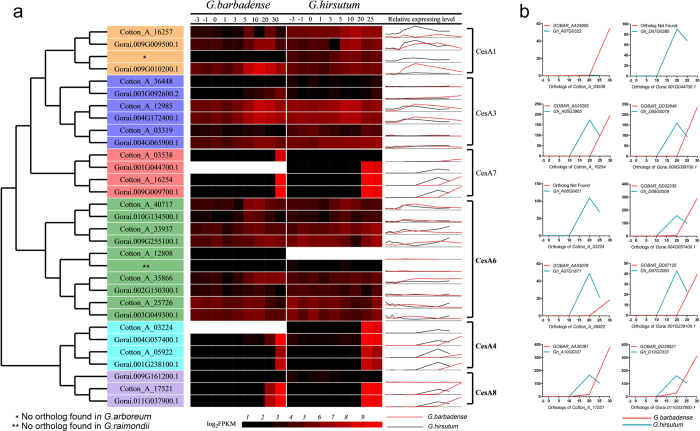
Cotton CesA genes and their expression in developing fiber cells of *G. barbadense* and *G. hirsutum.* (**a**) CesA genes from four cotton species were clustered (left) *via* MAGE5 using the maximum likelihood method. *G. arboreum* (Cotton_A) and *G. raimondii* (Gorai) contain 14 and 15 CesA genes, respectively, which are shown in the left column. The heat map (middle) shows the transcript level (FPKM, Reads Per Kilobase of exon model per Million mapped reads) of each homeologous gene in *G. barbadense* and *G. hirsutum* (Table 2) fibers at different DPA. The relative expression level in the two allotetraploid cottons was compared (right). CesA1, CesA3 and CesA6 are implicated in primary cell wall biosynthesis, and CesA4, CesA7 and CesA8 are implicated in secondary cell wall biosynthesis. (**b**) Temporal expression patterns of secondary cell wall *CesA* genes (CesA7, CesA4 and CesA8 clades) in *G. barbadense* and *G. hirsutum* fiber. Note that the expression was generally delayed in *G. barbadense* fiber. X-axis: day post-anthesis. Y-axis: FPKM.

Cellulose, which consists of linear chains of β (1–4)-linked D-glucose, is the major component of higher plant cell walls and the most abundant biopolymer on land. Plants express multiple cellulose synthases (CesAs) that, together with CesA-associated proteins, form the cellulose synthase complex^42^,^43^. Cotton fiber is distinct not only in its extensive elongation (ELS cotton fiber is longer than 35 mm) but also in its exceptionally high amount of cellulose, which constitutes more than 95% of the dry weight of mature fiber^16^,^44^. Notably, the first higher plant cellulose synthase gene was cloned from cotton^45^. Ten, 14 and 15 CesA genes are expressed in *Arabidopsis thaliana*^42^,^43^, *G. arboreum*^12^ and *G. raimondii*^10^, respectively (Fig. 5 and Table 2). We identified 29 CesA genes, including 14 A_t_ and 15 D_t_, in the *G. barbadense* genome, whereas 30 (14 A_t_ and 16 D_t_) CesA genes were identified in *G. hirsutum*; most CesA genes had been retained after the merger of the A and D genomes (Table 2 and Supplementary Fig. 13). Compared to *Arabidopsis*, each cotton genome or subgenome contains more genes in the CesA3, CesA4, CesA7 and CesA8 clades. Notably, chromosome 5 of both the A_t_ and D_t_ subgenomes of *G. barbadense* (GOBAR_AA25282, GOBAR_AA25287/GOBAR_DD32643, GOBAR_DD32648 and GOBAR_DD32650) and *G. hirsutum* (Gh_A05G3959, Gh_A05G3965, Gh_A05G3967/Gh_D05G0077, Gh_D05G0079 and Gh_D05G0084) as well as *G. arboreum* and *G. raimondii* contain a CesA cluster composed of 3 or, rarely, 2 genes, in addition to the CesA-like (CSL) genes (Table 2); thus, the duplication(s) occurred in the ancient cotton genome.

Although not exclusively, plant CesAs have functionally diverged into two major classes responsible for either primary cell wall or secondary cell wall biosynthesis^42^,^43^. Whereas spinnable cotton fiber evolved in the A-genome species and further developed in AD allotetraploids, the CesA gene family has not undergone expansion in any of the three cultivated cotton species sequenced. However, cotton fiber expresses many (at least 25) CesA genes (Fig. 5), demonstrating an enrichment of cellulose synthases in fiber cells. A comparison of the two allotetraploid cottons revealed that the secondary cell wall genes CesA4, CesA7 and CesA8 showed a delayed (>5 days) and more drastic up-regulation in *G. barbadense* fiber than in *G. hirsutum* fiber (Fig. 5), which indicates a prolonged duration of fiber elongation and a high activity of cellulose biosynthesis in the secondary cell wall formation stage. Additionally, this temporal expression pattern suggests that the functional allocation of CesA members to primary and secondary wall biosynthesis, which is primarily based on *Arabidopsis* research^42^,^43^,^46^, are likely conserved in angiosperms. Thus, both the retention of CesA family members and the expression pattern of functionally specialized genes in *G. barbadense* support the formation of extra-long and high-grade cotton fiber.

### Terpene synthases and the evolution of cotton phytoalexins

Terpenoids constitute a large family of natural compounds and play diverse roles in plant-environment interactions. Cotton plants accumulate a specialized group of cadinene-type sesquiterpenoids (including gossypol) that function as phytoalexins against pathogens and pests^47^,^48^. However, these sesquiterpenoids also reduce the value of cotton seeds that are rich in oil and proteins. Terpene synthases (TPSs) are a family of enzymes responsible for the synthesis of various terpenes from the 10-, 15-, and 20-carbon precursors assembled from the 5-carbon building blocks of IPP and its isomer DMAPP^49^. A manual search of the *G. barbadense* genome with TPS N- and C-terminal domains (PF01397 and PF03936) identified 115 TPS genes, including 44 monoterpene, 59 sesquiterpene and 8 diterpene synthases, as well as 4 triterpene (squalene) synthases. This number is higher than that in *T. cacao* (43), *Arabidopsis thaliana* (34) and *Vitis vinifera* (98) and similar to that in *G. hirsutum* (110) but slightly less than twice that in *G. raimondii* (69).

**Figure 6:**
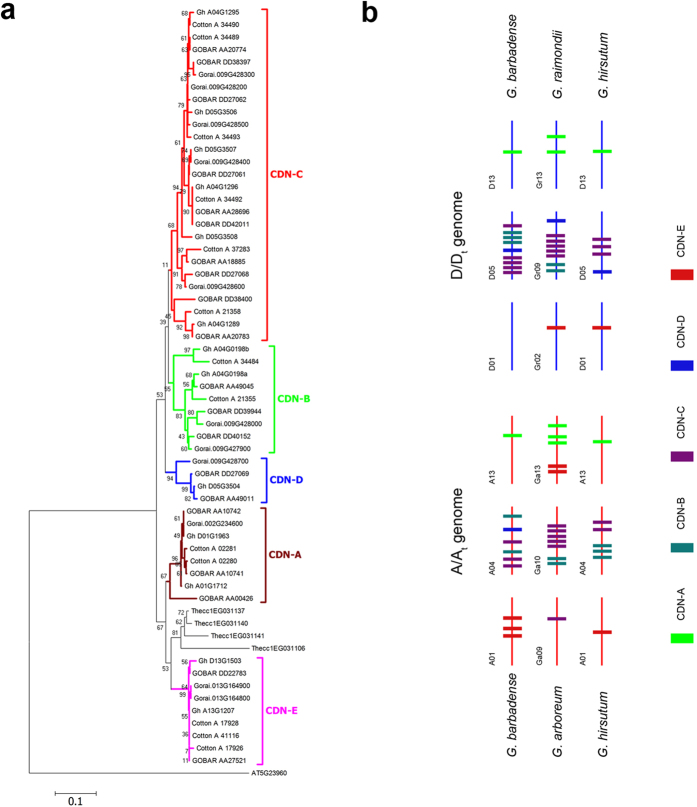
Phylogenetic analysis of (+)-δ-cadinene synthase (CDN) family genes and their genome distribution. (**a**) The amino acid sequences of CDNs of *G. arboreum* (Cotton_A), *G. raimondii* (Gorai), *G. hirsutum* (Gh) and *G. barbadense* (GOBAR) and *T. cacao* (Thecc) were used to build the phylogenetic tree using a neighbor-joining algorithm *via* the MEGA software. The *Arabidopsis thaliana* sesquiterpene synthase gene At5g23960 was used as a phylogenetic outgroup. (**b**) Chromosomal locations of the CDN genes in four *Gossypium* species as indicated.

The cotton sesquiterpene synthase (+)-δ-cadinene synthase (CDN) catalyzes the first step of gossypol biosynthesis^50^. The *G. barbadense* genome harbors 19 *CDN* family genes (sharing >80% nucleotide identity), whereas *G. raimondii*, *G. arboreum* and *G. hirsutum* harbor 11, 14 and 13 of these genes, respectively (Fig. 6 and Supplementary Table 18). These genes evolved faster than cotton speciation; thus, the *CDN* family evolved approximately 60 Mya based on the phylogenetics of cotton plants (Fig. 1). The *CDN* subfamilie*s* A and E were found closer to the ancient type and duplicated after the divergence of the cotton and cacao lineages (Fig. 6 and Supplementary Fig. 14). The variable *CDN* gene numbers in cotton species possibly refer to recent small-scale duplication events, *e.g.*, *CDN-A* member duplication in the D genome ~1 Mya (Supplementary Table 18 and Supplementary Fig. 14). Thus, the *CDN* subfamilies in *Gossypium* represent an example of the rapid lineage-specific evolution of critical genes for specialized metabolites.

## Discussion

ELS cotton likely produces one of the most resilient fibers in the plant kingdom; they are highly elongated and contain nearly pure cellulose. This draft sequence of the *G. barbadense* genome provides valuable genomic resources for studying various aspects of cotton. This draft sequence also facilitates breeding practices aimed at improving cotton fiber traits and increasing the production of high-quality biomass (cellulose).

The genomes of two or more parental species have combined to significantly change the genome structure and function of allopolyploid plants^38^,^51^,^52^. Inter-genomic chromosomal rearrangements, differential gene loss (the loss of some duplicates), gene conversion, divergence and the functional diversification of duplicated genes often arise with the onset of polyploidization^53^. Our comparative analysis of cotton genomes also provides new insight into dynamic allopolyploidy processes, such as the mechanism *via* TE (LTR retrons) bursts and pseudogenization, which have significantly contributed to plant genome evolution and trait formation.

## Methods

### Plant materials

Young leaves of *Gossypium barbadense* cv. Xinhai21, *G. arboreum* cv. Qingyangxiaozi and *G. raimondii* were collected from a single plant of each species for genomic DNA extraction and sequencing. For transcriptome sequencing, 28 samples from *G. barbadense* roots, stems, flowers, leaves, ovules and fibers were collected for total RNA extraction (Supplementary Table 19).

### DNA isolation, library construction and sequencing

Genomic DNA was isolated from fresh cotton leaves using a previously described method^54^. The shotgun library (300–800 bp fragments) was prepared from 5 μg of DNA using a standard protocol, and a total of 55,296,227 reads with an average length of 542 bp were produced *via* Roche 454 GS FLX to provide a 12-fold coverage of the genome. The paired-end libraries of different insertion sizes were constructed, and 1,325,215,140 pairs of 100-bp reads were produced *via* Illumina Hiseq2000 (Illumina, San Diego, CA) to provide 105-fold coverage of the genome. The 3-, 5-, 8 and 20-kb mate-pair libraries were constructed by combining the GS FLX and Illumina mate-pair protocol, and a total of 773,715,534 mate-pair reads were produced *via* Illumina Hiseq2000 to provide 61.9-fold sequencing coverage. The BAC library (insert, 80–120 kb) was constructed using the pCC1BAC vector (Epicentre Inc.) and Hind III enzyme digestion. The BAC clones were both-end sequenced using ABI 3730, and 20 BACs at a time were pooled and sequenced on Illumina Hiseq2000 to generate a 300-bp paired-end library.

For the PacBio library construction and sequencing, genomic DNA was sheared using a Covaris g-TUBE followed by purification *via* binding to pre-washed AMPure XP beads (Beckman Coulter Inc.). After end-repair, the blunt adapters were ligated, followed by exonuclease incubation to remove all un-ligated adapters and DNA. The final “SMRT bells” were annealed with primers and bound to the proprietary polymerase using the PacBio DNA/Polymerase Binding Kit P4 (Part Number 100–236–500) to form the “Binding Complex”. After dilution, the library was loaded onto the instrument with DNA Sequencing Kit 2.0 (Part Number 100–216–400) and a SMRT Cell 8Pac for sequencing. A primary filtering analysis was performed with the RS instrument, and the secondary analysis was performed using the SMRT analysis pipeline version 2.1.0.

### Genome assembly

The genomes of two diploid cotton species, *G. arboreum* and *G. raimondii*, were each sequenced at 100-fold coverage using Illumina Hiseq2000. The assembly resulted in 3,767,593 contigs of 1.5 Gb for *G. arboreum* and 1,111,300 contigs of 788 Mb for *G. raimondii*. These contigs, together with the published genomic data of *G. raimondii*^10^ and *G. arboreum*^12^, were used as template for grouping of *G. barbadense* sequencing reads into subgenomes, which resulted in totally 44.9% of the reads being A_t_-unique, 26.9% being D_t_-unique and 9.7% being both sharing. The remaining 18.5% none hit reads were further grouped during subgenome during sequence assembly.

After subgenome grouping, the A_t_ and D_t_ subgenomes of *G. barbadense* were assembled separately using a combined strategy. The Roche 454 reads were first assembled using Newbler v2.3. In total, 773,548 contigs with an average length of 2.5 kb were produced. Illumina pair-end reads, mate-pair reads, PacBio SMRT reads and BAC ends were then successively mapped to the contigs to improve quality. The 59,868 contigs (BACtigs) with an N50 of 23.8 kb from 515 BAC pools were merged. These approaches resulted in 4,586 A_t_ scaffolds and 2,186 D_t_ scaffolds with a total size of 2.2 Gb and maximum length of 3.4 Mb. Data statistics are given in Supplementary Table 2 and Table 1.

Finally, a high-density genetic map of *G. hirsutum* cv. TM-1 × *G. barbadense* cv. Hai7124 containing 4,999,048 SNPs^22^ was mapped to the *G. barbadense* assembly using the BWA program, which anchored 1.95 Gb or 88% of the assembled sequences and produced 26 pseudo-molecules (chromosomes).

### Gene prediction and annotation

Three gene prediction programs, GeneMark (v2.3a)^54^, Augustus (v2.5)^55^ and FgeneSH^56^, were used to predict protein-coding genes in the *G. barbadense* genome. A final gene model was produced by combining the three prediction results with an in-house developed program (GLAD), a tool that creates consensus gene lists by integrating evidence from homology, *de novo* prediction, and RNA-Seq/EST data. Annotation was performed by comparing the predicted proteins with non-redundant proteins (nr) and the UniProt and KEGG databases. Blast2go^57^ was used to assign preliminary GO terms to the predicted gene models. Transcription factors were predicted using PlantTFDB v3.0^58^. Protein domain predictions were performed using RPS-BLAST with a coverage >90%. The metabolic pathways were constructed using the KEGG database^59^.

### Ortholog identification and *Ks* calculation

Genes were classified into ortholog groups with OrthoMCL^60^ against OrthoMCL proteins (default parameters) [PMID: 12952885]. The orthologs between species, or homoeologs between the A_t_ and D_t_ subgenomes of *G. barbadense*, were defined using BLASTP based on the Bidirectional Best Hit (BBH) method with a sequence coverage >30% and identity >30%, followed by selection of the best match. The *Ka* and *Ks* between orthologs were calculated using the KaKs_Calculator^61^ *via* model averaging. The unique gene in each subgenome was defined using the following parameters: 1. protein sequence with no match according to BLASTP against proteins of the other subgenome with E-value 1E-3; and 2. the sum of the length of the high-scoring segment pairs (HSP) was less than 1/3 of the CDS length (*via* BLASTN) against the genome sequence of the other subgenome.

### Repeat and LTR retrotransposon analysis

Repetitive sequences were identified using RepeatScout with default parameters. The consensus sequences of each repeat family were used to identify repeat compositions in the genome *via* Censor. The complete LTR retron structures were predicted using LTR_finder^62^, and miniature inverted-repeat transposable elements (MITEs) were identified using MITE-Hunter^63^. Individual LTR retrotransposons were clustered into the same family using the 80–80–80 rule: If two TIR sequences share 80% or higher similarity in at least 80% of their length with an alignment length longer than 80 bp, the two sequences were clustered into the same family^64^.

The insertion ages of each full-length LTR retron were calculated based on the divergence between the left and right solo-LTR sequences using distmat from EMBOSS with the Kimura-2-parameter distance option, and insertion ages were calculated according to the formula T = K/(2r) (K = Kimura distance value, average substitution rate r = 2.6 × 10^–9^ in cotton).

### Pseudogene identification

Pseudogenes were predicted using Pseudopipe^65^ with default parameters. The predicted protein-coding gene sequences from both *G. barbadense* subgenomes were used as queries to search repeat-masked intergenic regions. Putative pseudogenes were filtered by excluding genes that significantly overlapped with functional gene annotations, genes with parental genes annotated as transposon elements or plastid genes, and genes with sequence lengths shorter than 150 bp.

### RNA extraction and transcriptome sequencing

The total RNA from each sample was extracted using TRIzol reagent (Invitrogen) following a standard protocol. The mRNAs were purified with the MicroPoly(A) Purist Kit (Ambion), fragmented and converted into an RNA-Seq library using the mRNAseq library construction kit (Illumina Inc.) and sequenced *via* Illumina Hiseq2000. The mRNAs of 28 samples were also pooled and sequenced on the 454 Genome Sequencer FLX instrument.

Sequence reads from all samples were cleaned using the FASTX toolkit (http://hannonlab.cshl.edu/fastx_toolkit/). All reads containing ‘N’ were discarded. Adapter sequences were then removed using the fastx_clipper program, followed by the removal of low-quality (Q < 5) bases from the 3′ end with fastq_quality_trimmer while requiring a minimum sequence length of 50 bp.

The RNA-Seq reads of each sample were mapped to the A_t_ and D_t_ genes using bowtie2^66^ with a mismatch in seed alignment of 0. Differentially expressed genes were identified *via* the DEGseq package using the MARS method (MA-plot-based method with Random Sampling model)^67^ based on their RPKM (Reads Per Kilobases per Million reads) or FPKM (reads per kilobase of exon model per million mapped reads) values^68^ with an FDR≤0.001 and |log_2_ Ratio |≥ 1 as the threshold. KEGG pathway enrichment was performed with a corrected P-value of < 0.05 as a threshold. GO enrichment was performed using Blast2go^57^.

## Additional Information

**Accession numbers**: The *G. barbadense* genome assembly contigs and scaffolds have been deposited in GenBank under PRJNA251673. The sequences and functional annotation of *G. barbadense* protein encoding genes, including predicted genes and transcriptome data, are available from the website. (http://database.chgc.sh.cn/cotton/index.html).

**How to cite this article**: Liu, X. *et al.* *Gossypium barbadense* genome sequence provides insight into the evolution of extra-long staple fiber and specialized metabolites. *Sci. Rep.* **5**, 14139; doi: 10.1038/srep14139 (2015).

## Supplementary Material

Supplementary Tables

Supplementary Information

## Figures and Tables

**Table 1:** Statistics of *G. barbadense* genome features.

**Category**	**A_t_**	**D_t_**
Genome Size (bp)	1,394,663,696	775,997,401
Gene Number	40,502	37,024
Ave. Gene Size (bp)	2,601	2,553
Total Gene Region	123,247,562	104,783,505
Ave. CDS Size (bp)	1,099	1,111
Max. CDS Length	19,647	16,596
Total Coding Region (bp)	52,095,402	45,586,340
Total Exon Number	240,755	208,290
Exon Number per Gene	5	5
Ave. Exon Size (bp)	216	219
Max. Exon Size (bp)	5,651	6,031
Total Intron Number	193,370	167,253
Ave. Intron Size (bp)	368	354
Max. Intron Size (bp)	85,091	86,599

**Table 2:** Cellulose synthase (CesA) genes in four cotton species.

Homologs in *G. arboreum*and *G. raimondii*	**Homologs in *G. hirsutum***	**Homologs in *G. barbadense***
	Genes related to the synthesis of cellulose in prototypical primarycell walls (CESA1, CESA3, CESA6 clades)	
CESA1 Clade		
no apparent ortholog	Gh_A05G3959	no apparent ortholog
Gorai.009G010200.1	Gh_D05G0077	GOBAR_DD32650
Cotton_A_16257	Gh_A05G3967	GOBAR_AA25287
Gorai.009G009500.1	Gh_D05G0084	GOBAR_DD32643
CESA3 Clade		
Cotton_A_03319	Gh_A08G0498	GOBAR_AA12453
Gorai.004G065900.1	Gh_D08G0584	GOBAR_DD11497
Cotton_A_12985	Gh_A08G1305	GOBAR_AA08823
Gorai.004G172400.1	Gh_D08G1597	GOBAR_DD05460
Cotton_A_36448	Gh_A02G1066	GOBAR_AA03569
Gorai.003G092600.2	Gh_D03G0611	GOBAR_DD02554
CESA6 Clade		
Cotton_A_25726	Gh_A02G1317	GOBAR_AA16276
Gorai.003G049300.1	Gh_D03G0455	GOBAR_DD10475
Cotton_A_33937	Gh_A05G3694	GOBAR_AA32700
Gorai.009G255100.1	Gh_D05G2313	GOBAR_DD35549
Cotton_A_40717	Gh_A06G1017	GOBAR_AA04815
Gorai.010G134500.1	Gh_D06G1219	GOBAR_DD30509
Cotton_A_35866	Gh_A11G3209	GOBAR_AA22611
Gorai.002G150300.1	Gh_D11G2235	GOBAR_DD13415
Cotton_A_12808	no apparent ortholog	GOBAR_AA34523
no apparent ortholog	Gh_D12G0885	GOBAR_DD19420
	**Genes related to the synthesis of cellulose in prototypicalsecondary cell walls (CESA4, CESA7, CESA8 clades)**	
CESA4 Clade		
Cotton_A_05922	Gh_A07G1871	GOBAR_AA03078
Gorai.001G238100.1	Gh_D07G2083	GOBAR_DD07125
Cotton_A_03224	Gh_A08G0421	no apparent ortholog
Gorai.004G057400.1	Gh_D08G0509	GOBAR_DD02235
CESA7 Clade		
Cotton_A_03538	Gh_A07G0322	GOBAR_AA24905
Gorai.001G044700.1	Gh_D07G0380	no apparent ortholog
Cotton_A_16254	Gh_A05G3965	GOBAR_AA25282
Gorai.009G009700.1	Gh_D05G0079	GOBAR_DD32648
CESA8 Clade		
Gorai.009G161200.1	Gh_D05G1460	GOBAR_AA30803*
Cotton_A_17521	Gh_A10G0327	GOBAR_AA30381
Gorai.011G037900.1	Gh_D10G0333	GOBAR_DD29521

^*^May have translocated.
