# Supplementary material for: Gossypium barbadense genome sequence provides insight into the evolution of extra-long staple fiber and specialized metabolites
Source: Sci Rep. 2015 Sep 30;5:14139. doi: 10.1038/srep14139 (PMC4588572; doi:10.1038/srep14139)
Supplement: Supplementary Information [file srep14139-s2.pdf]

Gossypium barbadense genome sequence provides insight into the evolution of extra-long staple fiber and specialized metabolites

Xia Liu[1,†], Bo Zhao[2, †], Hua-Jun Zheng[3,†], Yan Hu[4,†], Gang Lu[3], Chang-Qing Yang[2], Jie-Dan Chen[4], Jun-Jian Chen[1], Dian-Yang Chen[2], Liang Zhang[3], Yan Zhou[3,5], Ling-Jian Wang[2], Wang-Zhen Guo[4], Yu-Lin Bai[1], Ju-Xin Ruan[2], Xiao-Xia Shangguan[2], Ying-Bo Mao[2], Chun-Min Shan[2], Jian-Ping Jiang[3], Yong-Qiang Zhu[3], Lei Jin[3], Hui Kang[3], Shu-Ting Chen[3], Xu-Lin He[3], Rui Wang[3], Yue-zhu Wang[3], Jie Chen[3], Li-jun Wang[3], Shu-Ting Yu[3], Bi-Yun Wang[3], Jia Wei[3], Si-Chao Song[3,] Xin-Yan Lu[3], Zheng-Chao Gao[3], Wen-Yi Gu[3], Xiao Deng[6,] Dan Ma[4], Sen Wang[4], Wen-Hua Liang[4], Lei Fang[4], Cai-Ping Cai[4], Xie-Fei Zhu[4], Bao-Liang Zhou[4], Z. Jeffrey Chen[4,8], Shu-Hua Xu[7], Yu-Gao Zhang[1,*], Sheng-Yue Wang[3,*], Tian-Zhen Zhang[4,*], Guo-Ping Zhao[2,3,5,*] & Xiao-Ya Chen[2,*]

[1]Esquel Group, 25/F Eastern Cenrtal Plaza, 3 Yin Hing Road, Shau Kei Wan, Hongkong, China.

[2]National Key Laboratory of Plant Molecular Genetics, National Plant Gene Research Center, Institute of Plant Physiology and Ecology, Shanghai Institutes for Biological Sciences, Chinese Academy of Sciences, Shanghai 200032, China.

[3]Shanghai–Ministry of Science and Technology Key Laboratory of Health and Disease Genomics, Chinese National Human Genome Center at Shanghai, Shanghai 201203, China.

[4]Nanjing Agricultural University, Nanjing, Jiangsu 210095, China.

[5]State Key Laboratory of Genetic Engineering, School of Life Sciences, Fudan University, Shanghai 200433, China.

[6]The Institutes of Biology and Medical Sciences, Soochow University, Suzhou, Jiangsu 214123, China.

[7]Max Planck Independent Research Group on Population Genomics, Chinese Academy of Sciences and Max Planck Society (CAS-MPG) Partner Institute for Computational Biology (PICB), Shanghai Institutes for Biological Sciences, Chinese Academy of Sciences, Shanghai 200031, China.

[8]Institute for Cellular and Molecular Biology and Center for Computational Biology and Bioinformatics, The University of Texas at Austin, Austin, Texas 78712, USA.

†These Authors contributed equally to this work

*Corresponding Author: xychen@sibs.ac.cn; gpzhao@sibs.ac.cn; cotton@njau.edu.cn; wangsy@chgc.sh.cn; zhangyu@esquel.com

Supplementary Figure 1

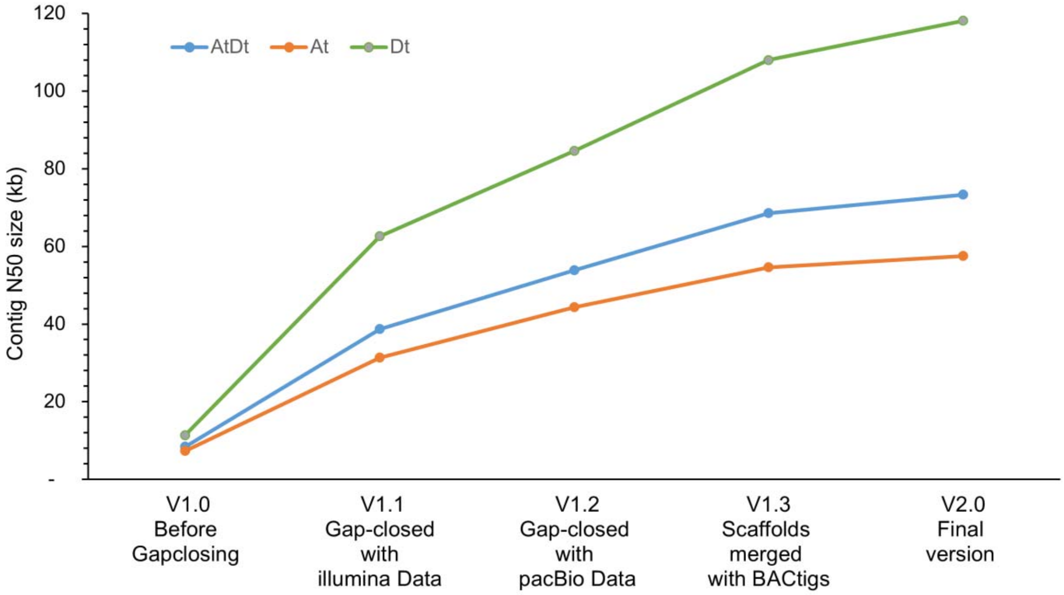

Supplementary Figure 1. Gap-closing efficiency of different types of reads. Starting with initial V1.0 assembly, multiple gap closing procedures were implemented to improve assembly continuity. Illumina paired-ends were used to close most small gaps in the starting version, then the PacBio SMRT sequencing data and contigs from BAC pools (BACtigs) were merged to close remaining gaps with relative bigger sizes.

Supplementary Figure 2

a

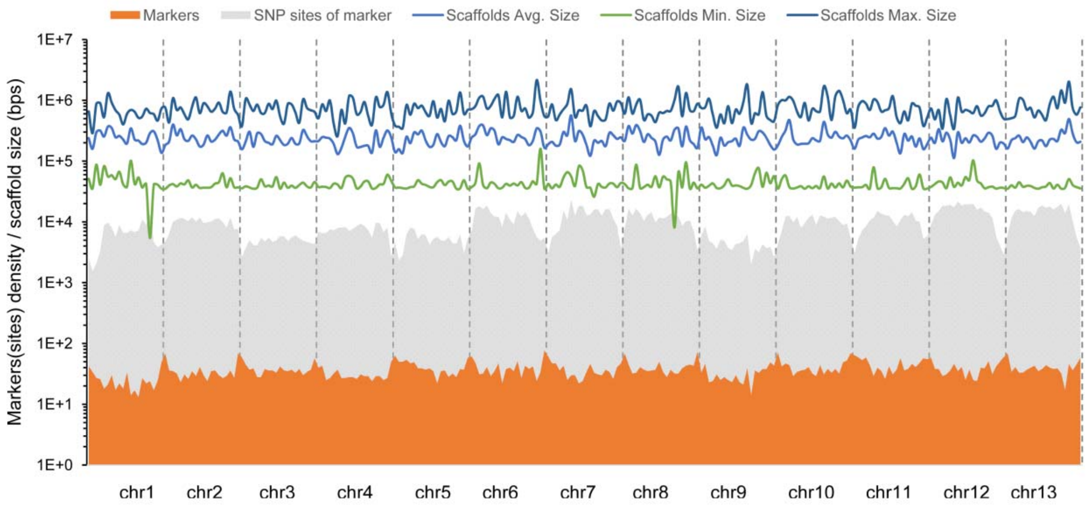

b

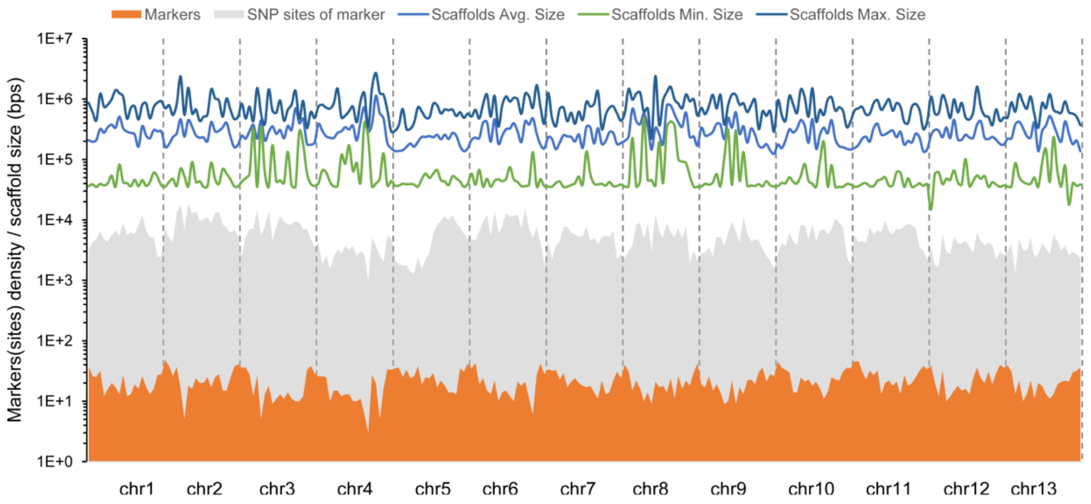

Supplementary Figure 2. Scaffolds coverage based on genetic maps of 13 A_t (a) and 13 D_t (b) chromosomes. Scaffold sizes across chromosomes are represented by threes lines, standing for max (indigo), average (blue) and minimal (green) scaffold sizes, respectively. The density of SNP sites and the corresponding genetic marks (bins of SNPs mapped to contigs) are demonstrated as gray plots and orange, respectively. Calculations were performed with window of 1/20 of chromosome size.

Supplementary Figure 3

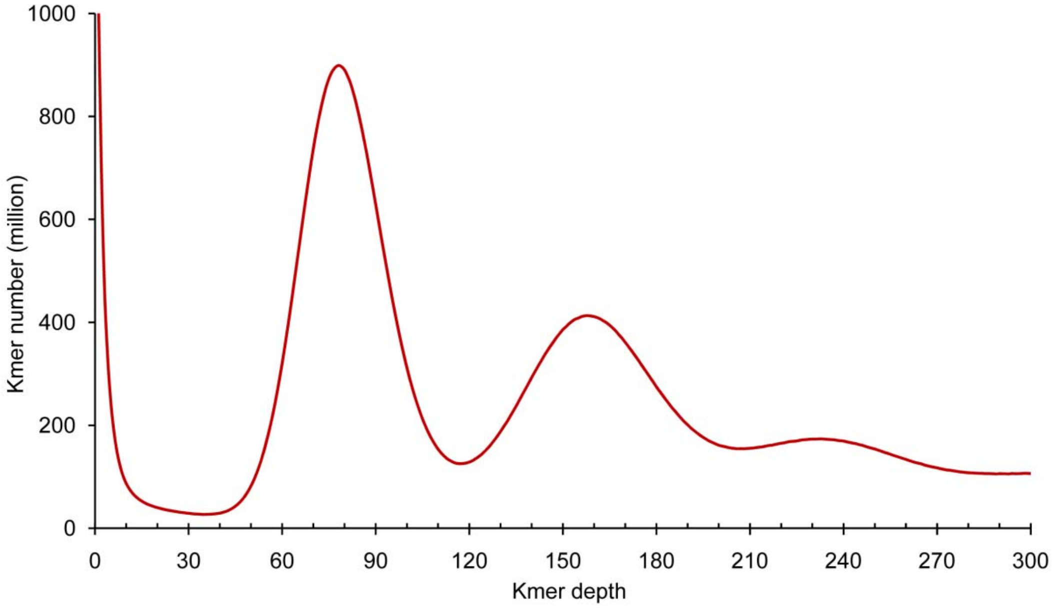

Supplementary Figure 3. 17-mer histogram analysis of *G. barbadense* reads. Data from two Illumina paired-end (PE) libraries with insert sizes of 180 bp and 300 bp were used to construct the histogram graph. Totally 390.3 Giga kmers were acquired, with observed average depth peak at 158 fold, adding up to 2.47 Gb genome size according to the equation of L/C = G (where L stands for k-mer number, C for average depth and G for genome size to be determined).

Supplementary Figure 4

a

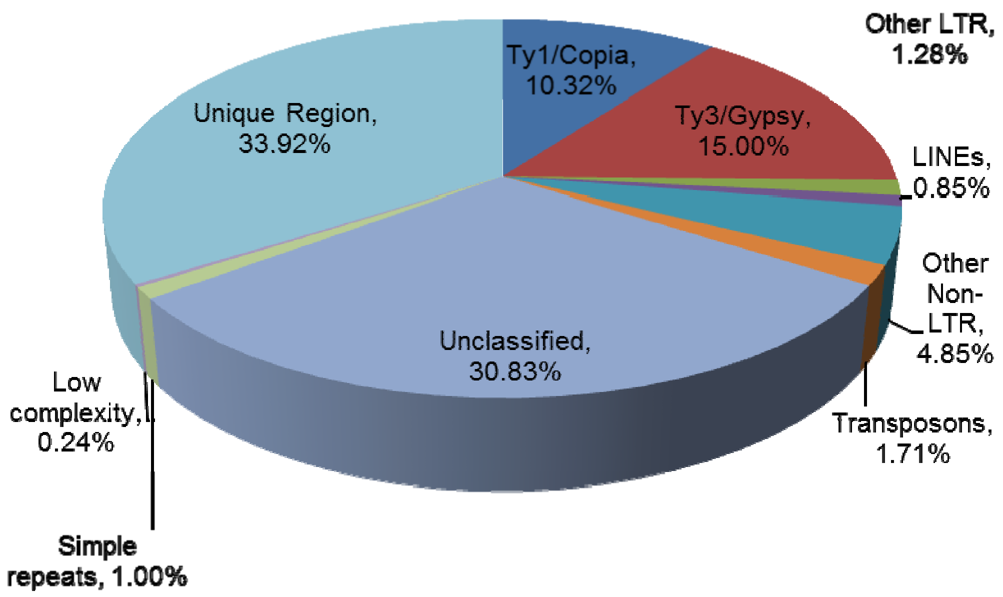

b

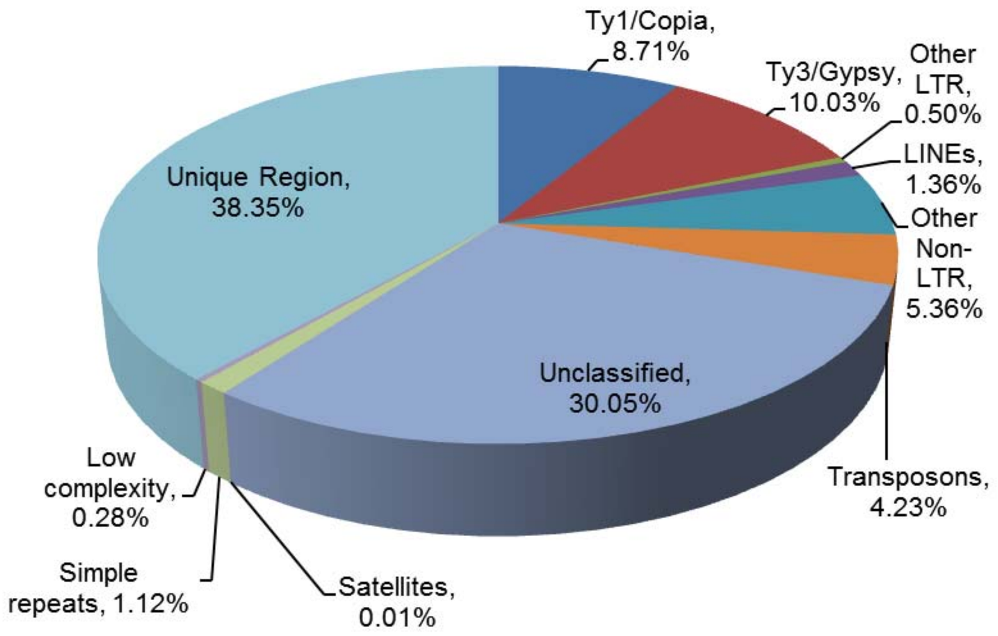

Supplementary Figure 4. Distribution of repeats in the *G. barbadense* genome. (**a**) A_t subgenome. (**b**) D_t subgenome. LTR retrotransposons are divided into Ty1, Ty3, Lines and other LTRs.

Supplementary Figure 5

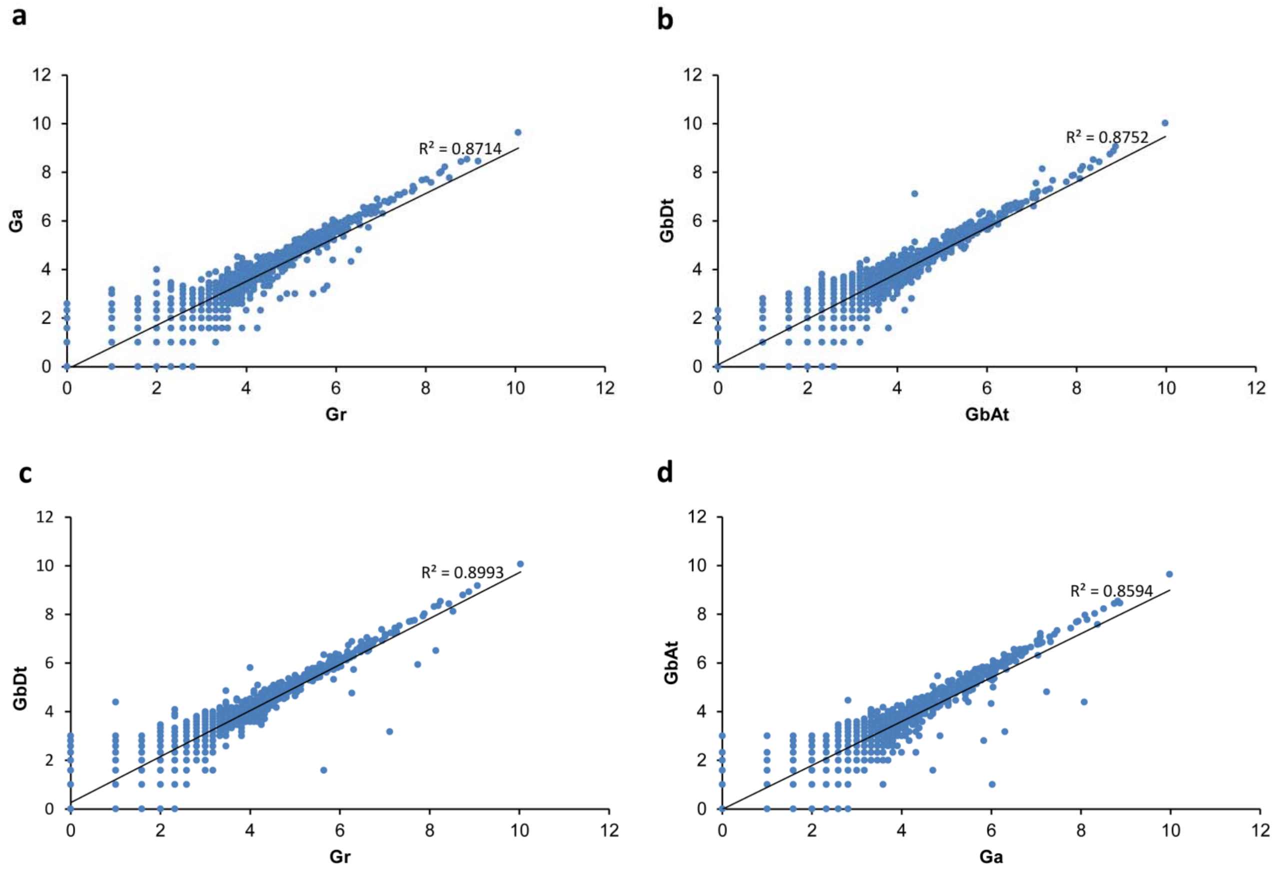

Supplementary Figure 5. **Relations of Pfam domain families among cotton genomes.** (**a**) Domain distribution between A and D genomes of diploid cotton species. (**b**) Domain distribution between A_t and D_t subgenomes in allotetraploid cotton *G. barbadense*. (**c**) Domain distribution between D genome of diploid cotton *G. raimondii* and D_t subgenome in *G. barbadense*. (**d**) Domain distribution between A genome of diploid cotton *G. arboreum* and A_t subgenome of *G. barbadense*. Each dot represents a Pfam domain family, and the number on the axes is $\log_2$ of the number of genes in each family. Ga: *G. arboreum*, Gb: *G. barbadense*, Gr: *G. raimondii*.

Supplementary Figure 6

a

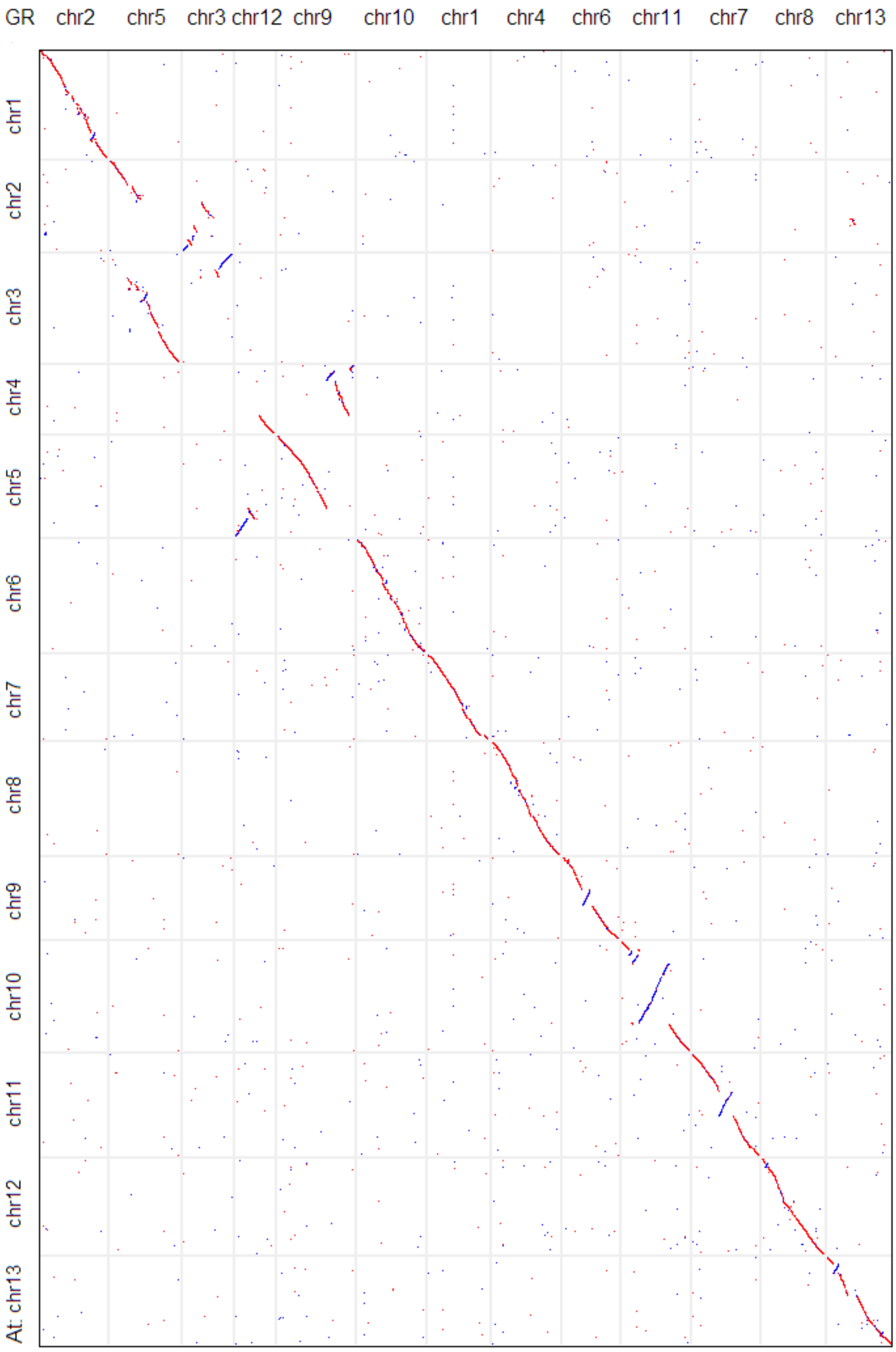

Supplementary Figure 6

b

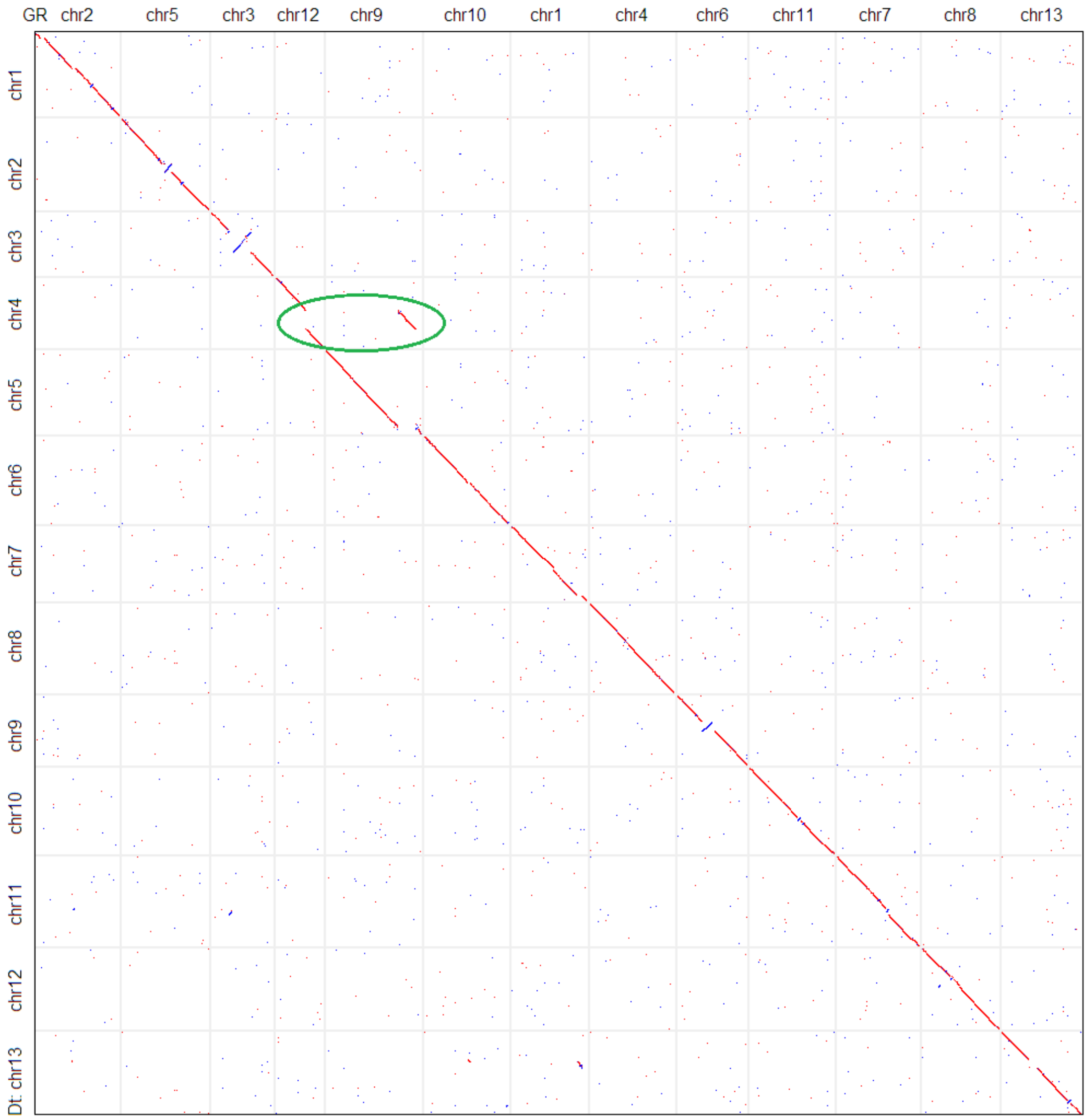

Supplementary Figure 6. Dot plots of diploid and tetraploid cotton genomes. Chromosome mapping between *G. raimondii* and *G. barbadense* A_t subgenome (**a**), and between *G. raimondii* and *G. barbadense* D_t (**b**). A_t01~A_t13, or D_t01~D_t13, of *G. barbadense* are placed from top to down on y-axis, while the corresponding chromosomes from *G. raimondii* are placed on x-axis. In graph, forward matchs are marked as red, and reversed as blue. A_t02/A_t03, A_t04/A_t05 reciprocal translocations are clearly shown in dot plots map (**a**), and an ancental translocation of D_t04/D_t05 caused a jump of synteny block is circled by ellipse (**b**). The dot plots were generated by alignment of nucleotide sequences, and each dot represents a portion of the genome or subgenome (eg., 1/1000).

Supplementary Figure 7

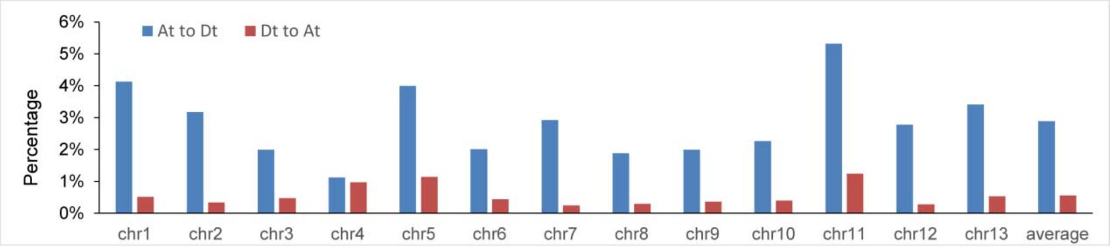

Supplementary Figure 7. Statistics of inter-subgenome translocations. Blue bar stands for D_t sequences translocated from A_t, whereas darkedred represents sequences translocated from D_t to A_t. Chromosome numbers are lined up in x-axis and the percentage of translocated region divied by total chromosome size is given in y-axis. According to data-grouping analysis, reads groups marked with A_t or D_t were seperately assembled into contigs and scaffolds, and scaffolds were then anchored to chromosomes by genetic markers. Only very small portion of sequences (~20-Mb) presented some contradictions between the assembly grouping and the genetic marker location, ie., a scaffold from A_t assemby had a D_t chormosome linked genetic marker, or *vice versa*. Despite the existence of sequencing error, mis-grouping sequences, low confidence SNP markers and other potential tranlocated regions were determined by realignment to two reference genomes (*G. aboreum* and *G. raimondii*). Sequences with higher similarity to one of these two subgenomes are believed to have originated from its corresponding progenitor of the subgenome, and, when conflicted, they may indicate the existence of inter-subgenome translocation.

Supplementary Figure 8

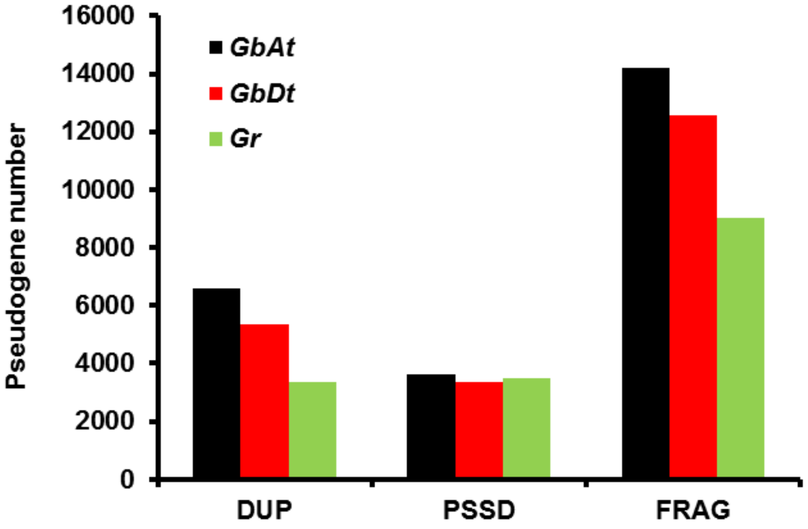

Supplementary Figure 8. Three categories of predicted pseudogenes. DUP: duplicated, PSSD: processed, FRAG: fragmented. Duplicated pseudogenes are commonly derived from gene duplication, processed pseudogenes are generated by the integration of reversed-transcribed cDNA into genomes, and fragmented pseudogenes are those that cannot be assessed as processed or duplicated. For reference, see Zhang et al., *Bioinformatics* **22**, 1437-1439 (2006).

Supplementary Figure 9

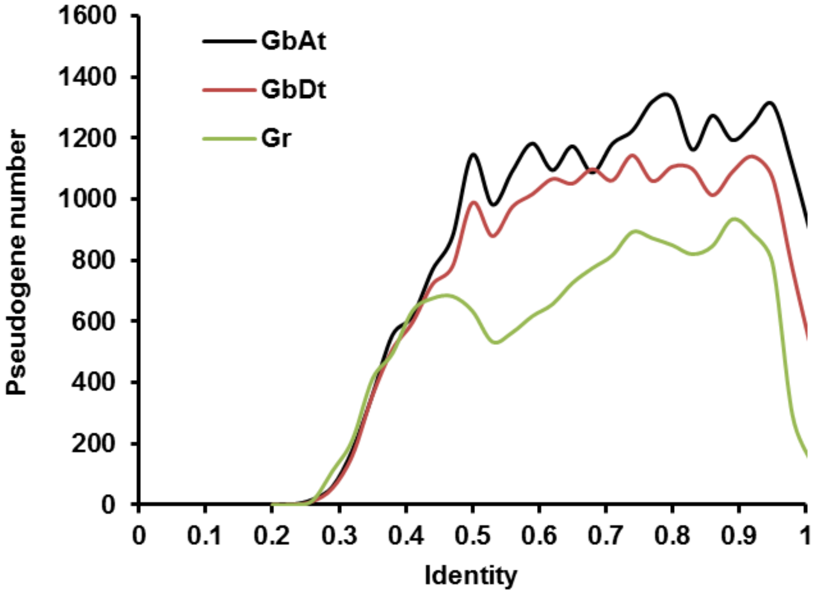

Supplementary Figure 9. Alignment of pseudogenes to their closest functional genes. Identity was calculated based on blastn between the pseudogenes and its parental genes. Gb: *Gossypium barbadense*, Gr: *Gossypium raimondii*.

Supplementary Figure 10

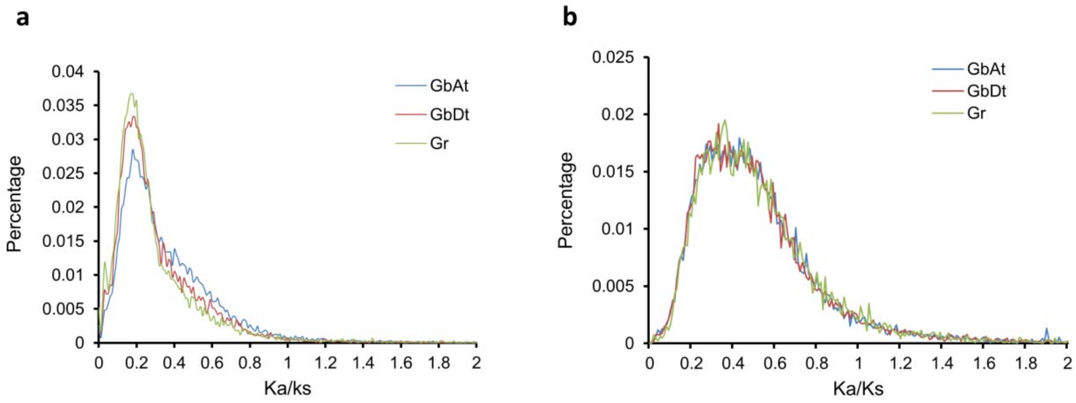

Supplementary Figure 10. Strength of purifying selection on predicted protein-coding genes and pseudogenes. (**a**) *Ka/Ks* distribution of functional gene paralogs. Data are grouped in 0.01 unit. (**b**) *Ka/Ks* distribution of pseudogenes and their closest functional paralogs. Data are grouped in 0.01 unit.

Supplementary Figure 11

a

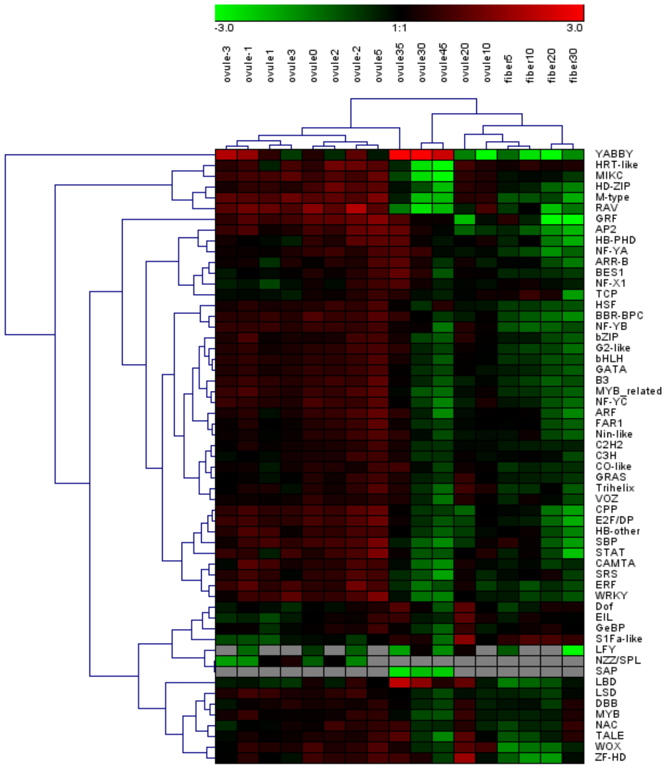

b

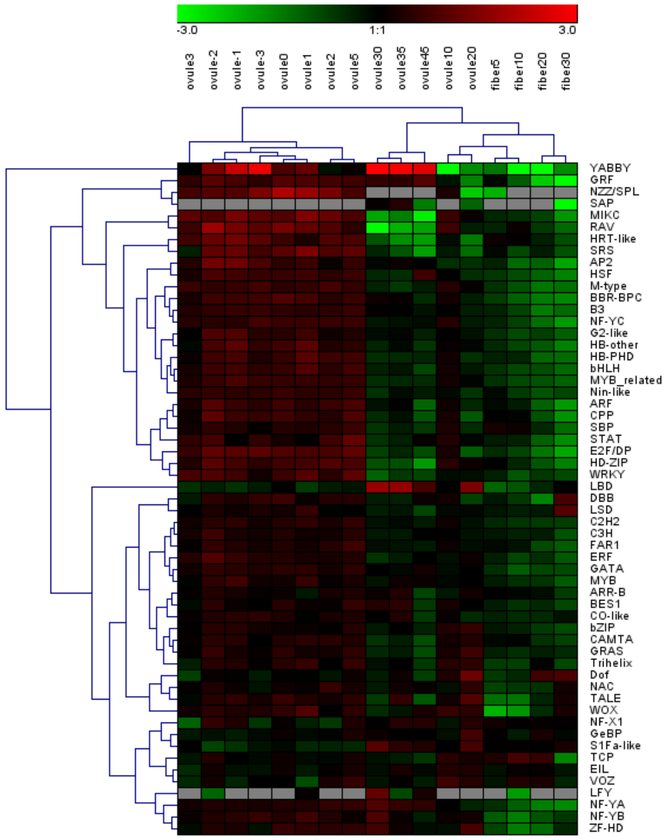

Supplementary Figure 11

c

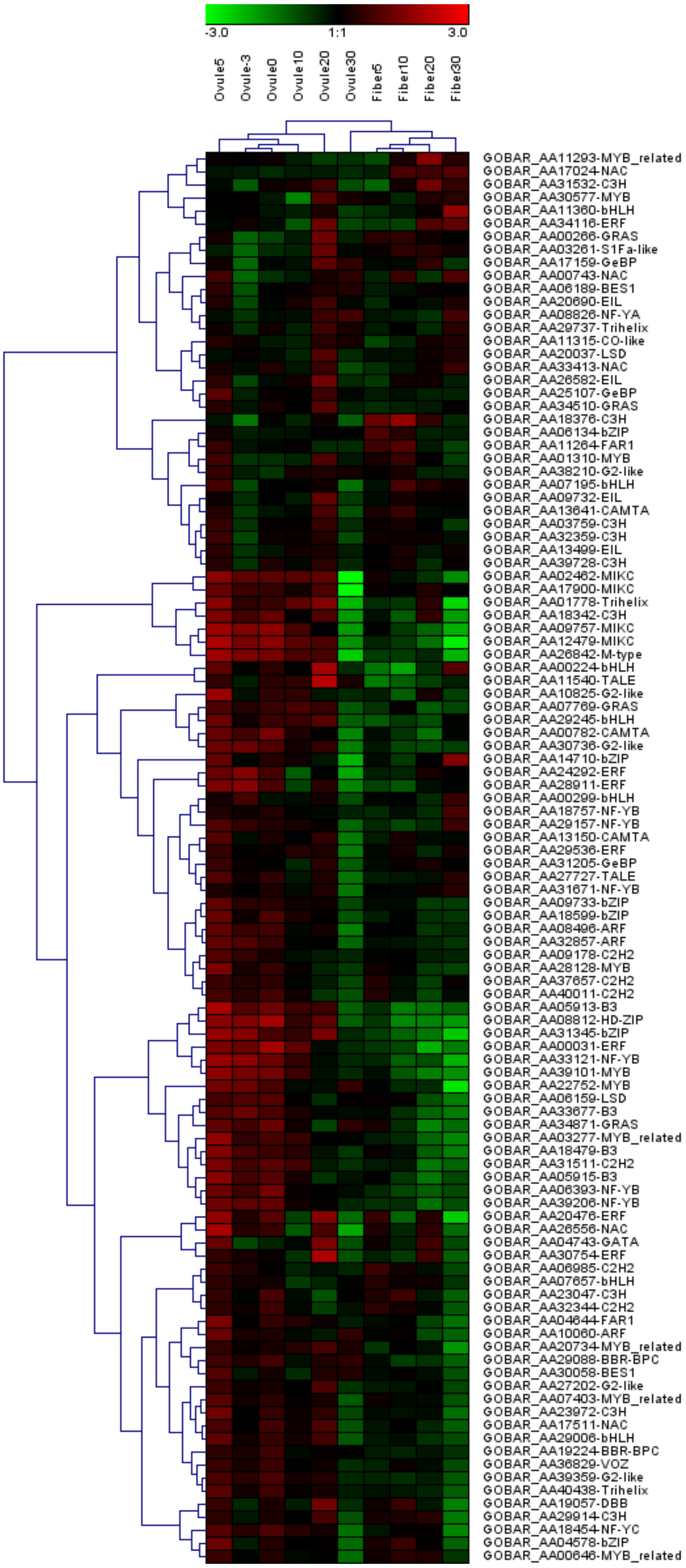

Supplementary Figure 11

d

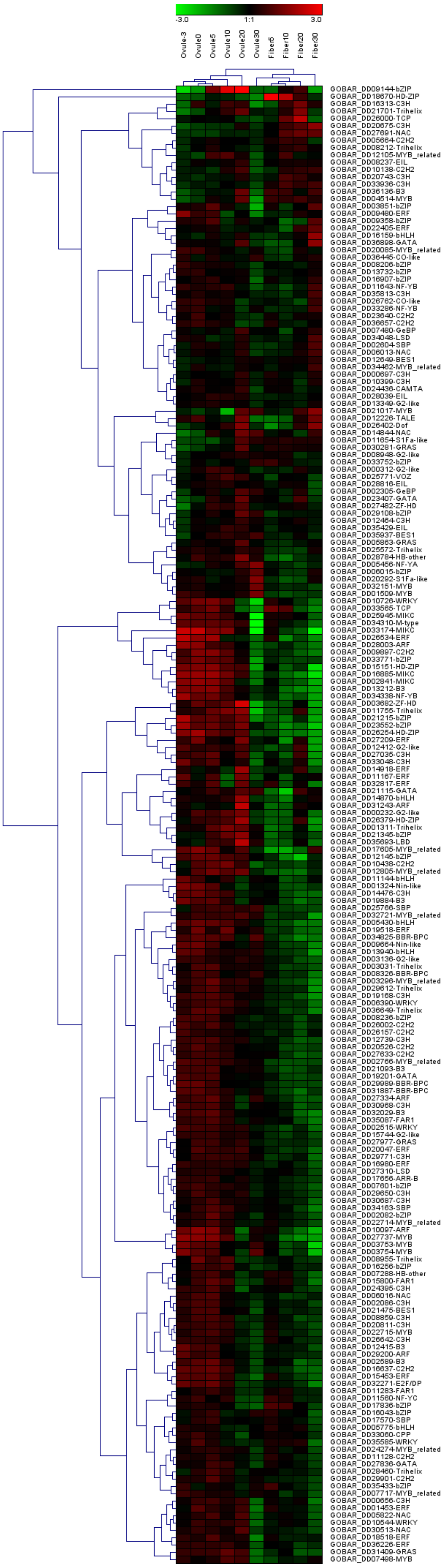

Supplementary Figure 11. Expression profiles of transcription factor (TF) families of A_t and D_t in ovules and fibers at different developmental stages. (**a**) Expression profile of TF families in A_t. (**b**) Expression profile of TF families in D_t. (**c**) Expression profile of highly expressed TF genes (RPKM>10) in $A_{t.}$ (**d**) Expression profile of highly expressed TF genes (RPKM>10) in $D_{t.}$ The expression level of each TF family is represented by the average RPKM values of all genes in this family. RPKM values (Log_2) of each family were clustered by complete linkage hierarchical clustering method. Green indicates lower expression, and red indicates higher expression.

Supplementary Figure 12

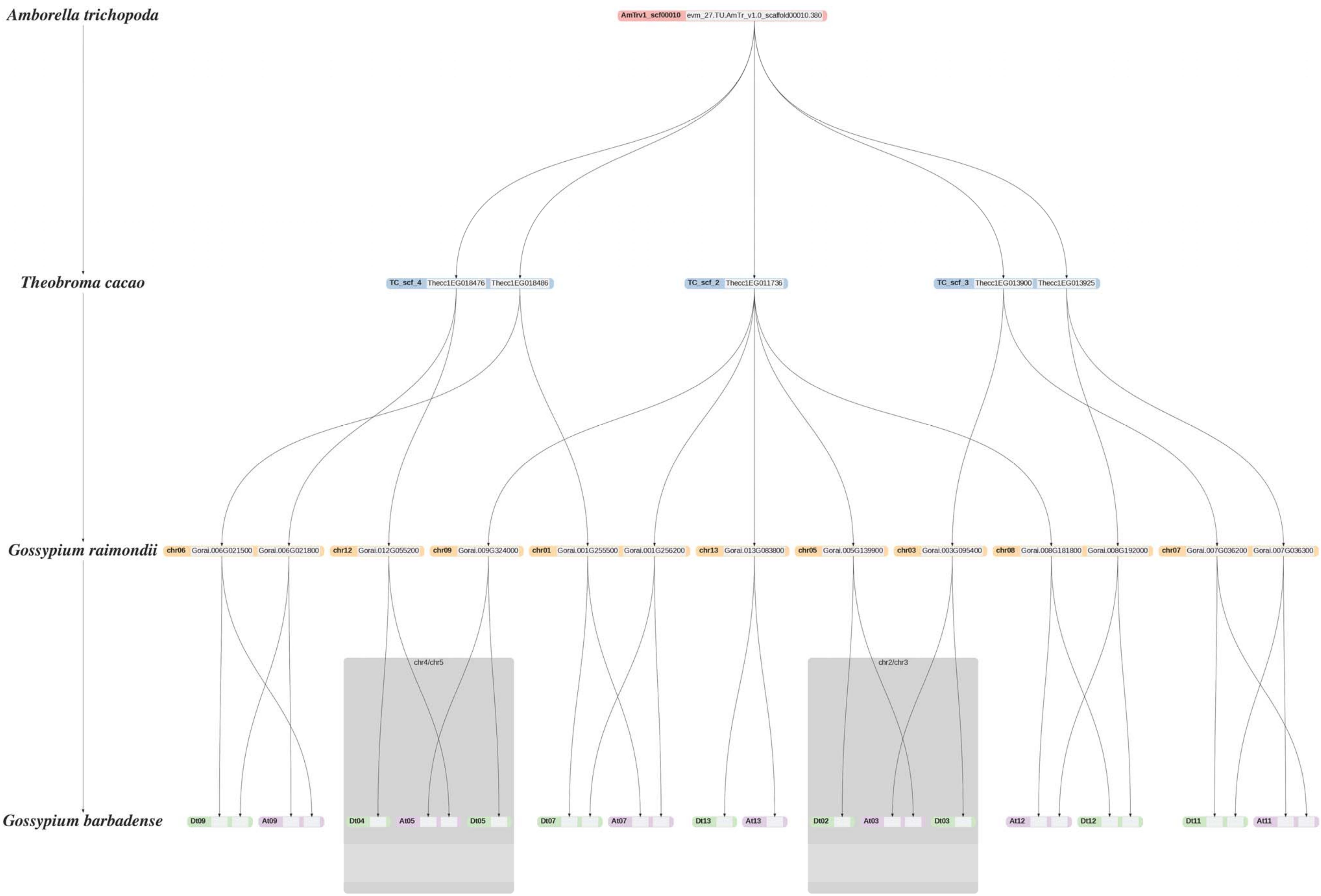

Supplementary Figure 12. Expansion and subsequent diversification of *PRE* family genes in *Gossypium.* The gene sets of *Amborella trichopoda*, *Theobroma cacao*, *G. raimondii*, *G. barbadense* were scanned with the well defined *Arabidopsis thaliana* PRE sequences (AT5G39860, AT5G15160, AT1G74500, At3g47710, AT3G28857, At1g26945) to distinguish a core PRE set in each lineage. The gene ancestry information (http://www.phytozome.net) was further used to ascertain the number of *PRE* genes present in each plant. Only one best-predicted homolog was revealed in *Amborella*, and we arbitrary define it as *Amborella PRE1* gene that represents here the most ancestral PRE member in angiosperms. Syntenic regions containing *PRE* genes were used to unravel the evolutionary history leading to the expansion and diversification of *Gossypium* PRE family. Two intra-subgenome reciprocal translocations (chr02/chr03 and chr04/chr05) carrying *PRE* genes are indicated with grey boxes.

Supplementary Figure 13

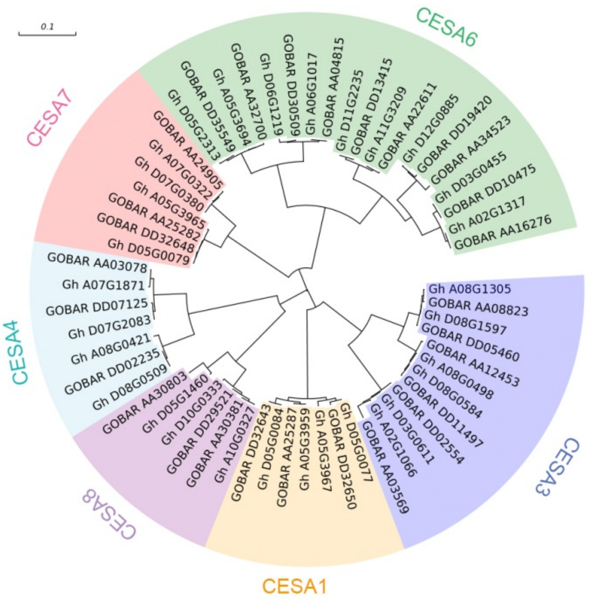

Supplementary Figure 13. Clustering analysis of *CesA* genes in *G. barbadense* and *G. hirsutum.* There are 29 cellulose synthase (CesA) genes *G. barbadense* genome (GOBAR), among which 14 in A_t and 15 in D_t subgenome, respectively, compared to 30 in *G. hirsutum* (Gh) with 14 in A_t and 16 in D_t. The ORFs were clustered by MAGE5 using maximum likelihood method.

Supplementary Figure 14

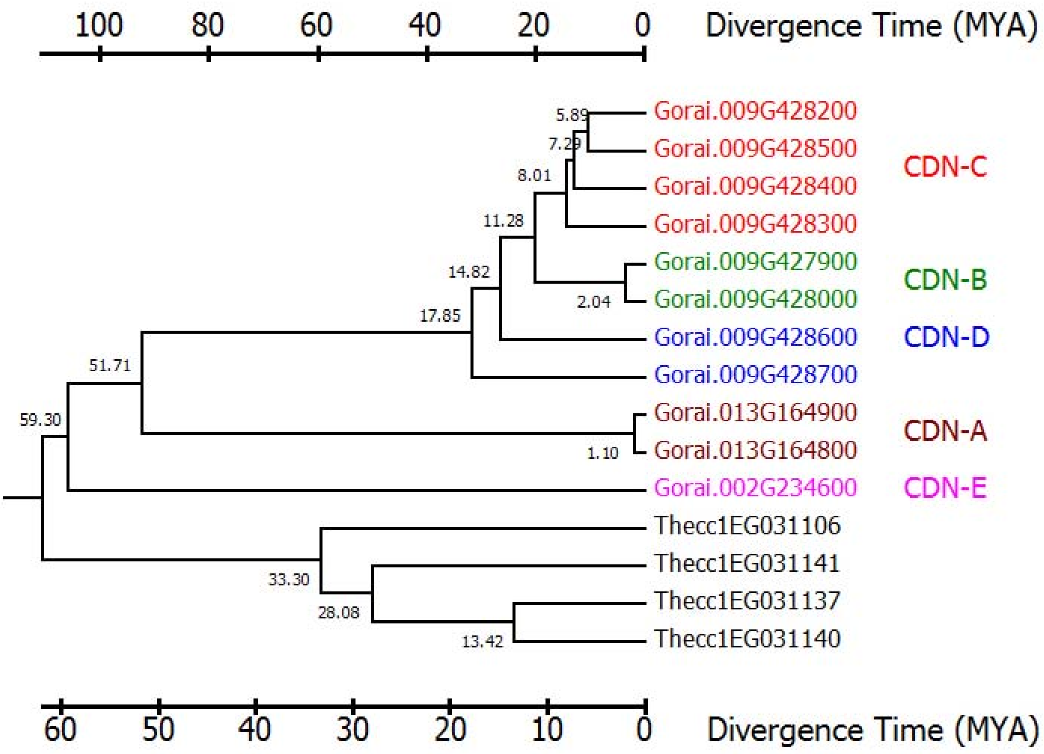

Supplementary Figure 14. Divergence of CDN genes of *G. raimondii* and *T. cacao*. CDN ORFs from *G. raimondii* and *T. cacao* genomes were clustered by neighbor joining algorithm and the divergence time was estimated according to the nucleotide substitution rates (upper scale bar), and adjusted according to the estimated divergence time of cotton linage (~ 60 Mya, lower scale bar) with MEGA software. Numbers at each branch node represent estimated time. See also Figure 1.

Supplementary Table 1. Grouping results of different sequencing libraries

Group	HiSeq 2000		454 GS FLX		Overall	
	Total bases	Ratio	Total bases	Ratio	Total bases	Ratio
A_t	1,066,173,459	44.7%	51,641,008	49.7%	1,117,814,467	44.9%
D_t	639,865,519	26.8%	29,981,752	28.9%	669,847,271	26.9%
Both	227,649,719	9.5%	14,542,252	14.0%	242,191,971	9.7%
None	452,467,973	19.0%	7,708,060	7.4%	460,176,033	18.5%
Total	2,386,156,670	100.0%	103,873,072	100.0%	2,490,029,742	100.0%

Supplementary Table 3. Repeats distribution in *G. barbadense* genome

Type	Number of elements		Length occupied (bp)		Percentage of sequence	
	A_t	D_t	A_t	D_t	A_t	D_t
LINEs:	11,626	20,267	11,861,690	10,577,775	0.85%	1.36%
LINE1	9,256	17,542	10,198,694	9,383,067	0.73%	1.21%
LINE2	292	1,828	233,216	716,566	0.02%	0.09%
L3/CR1	2,078	433	1,429,780	347,665	0.10%	0.04%
LTR elements	390,652	186,476	384,983,014	149,291,347	26.60%	19.24%
DNA elements	62,344	77,768	23,907,248	32,806,245	1.71%	4.23%
Simple repeats	207,969	145,029	13,918,742	8,700,108	1.00%	1.12%
Low complexity	58,220	38,444	3,345,799	2,140,612	0.24%	0.28%
Other repeats	947,402	818,875	485,715,903	264,231,571	34.83%	34.08%

Supplementary Table 5. *Ks* between genomes/subgenomes of Cotton

	Peak *Ks*	Divergence time (Mya)
GaA_2 and GrD_5	0.041	8.1
GbA_t and GaA_2	0.007	1.35
GbA_t and GbD_t	0.041	8.1
GbA_t and GhA_t	0.005	0.96
GbA_t and GhD_t	0.035	6.73
GbA_t and GrD_5	0.039	7.5
GbD_t and GhD_t	0.005	0.96
GbD_t and GaA_2	0.041	8.1
GbDt and GhAt	0.041	8.1
GbD_t and GrD_5	0.011	2.11
GhA_t and GaA_2	0.006	1.15
GhA_t and GhD_t	0.043	8.27
GhA_t and GrD_5	0.037	7.11
GhD_t and GaA_2	0.037	7.11
GhD_t and GrD_5	0.011	2.11

Ga, *G. arboreum*; Gb, *G. barbadense*; Gh, *G. hirsutum*; Gr, *G. raimondii*.

Supplementary Table 8. LTR retrons in different cotton genomes

Cotton	Number of LTR-retrons
G. raimondii (D_5)	2,992
G. arboreum (A_2)	8,620
G. barbadense A_t	6,014
G. barbadense D_t	2,422
G. hirsutum A_t	4,283
G. hirsutum D_t	2,731
G. hirsutum Unassigned	1,610

Supplementary Table 9. Summary of predicted pseudogenes in cotton genomes

Genome	DUP	FRAG	PSSD	Total	Closest parent	Size (Mb)	Identity above 90%
GbA_t	6,566	14,201	3,636	24,403	10,511	20.5	4,353
GbD_t	5,364	12,571	3,373	21,307	9,391	15.7	3,301
Gr	3,346	9,023	3,500	15,869	5,429	13.3	1,942

DUP, duplicated; FRAG, fragmented; PSSD, processed. See also **Supplementary Figure 8**.
Gb, *G. barbadense*; Gr, *G. raimondii*.

Supplementary Table 13. GO enrichment analysis of highly expressed genes in fiber

GO-ID	Term	Category	FDR	P-Value	#Test	#Ref	Over/Under
GO:0031224	intrinsic to membrane	C	3.37E-13	1.34E-16	133	1651	over
GO:0016021	integral to membrane	C	3.37E-13	1.49E-16	128	1560	over
GO:0016020	membrane	C	3.37E-13	1.98E-16	230	3653	over
GO:0044425	membrane part	C	1.96E-11	1.53E-14	146	2018	over
GO:0071944	cell periphery	C	2.38E-04	2.33E-07	62	817	over
GO:0006810	transport	P	6.77E-04	9.06E-07	121	2089	over
GO:0051234	establishment of localization	P	6.77E-04	9.27E-07	121	2090	over
GO:0051179	localization	P	7.58E-04	1.19E-06	122	2123	over
GO:0007018	microtubule-based movement	P	9.42E-04	1.70E-06	16	94	over
GO:0005975	carbohydrate metabolic process	P	9.42E-04	1.86E-06	62	873	over
GO:0005856	cytoskeleton	C	9.42E-04	2.03E-06	24	203	over
GO:0005794	Golgi apparatus	C	0.001303656	3.06E-06	20	151	over
GO:0005874	microtubule	C	0.001662286	4.88E-06	16	103	over
GO:0051258	protein polymerization	P	0.001671532	5.23E-06	11	47	over
GO:0044430	cytoskeletal part	C	0.001705131	5.67E-06	20	158	over
GO:0007017	microtubule-based process	P	0.001928969	6.79E-06	17	119	over
GO:0006825	copper ion transport	P	0.002578507	1.03E-05	7	16	over
GO:0032561	guanyl ribonucleotide binding	F	0.002578507	1.06E-05	28	290	over
GO:0005525	GTP binding	F	0.002578507	1.06E-05	28	290	over
GO:0019001	guanyl nucleotide binding	F	0.002765055	1.19E-05	28	292	over
GO:0004427	inorganic diphosphatase activity	F	0.003139539	1.41E-05	7	17	over
GO:0005886	plasma membrane	C	0.004749284	2.23E-05	45	612	over
GO:0006461	protein complex assembly	P	0.004853524	2.47E-05	16	119	over
GO:0070271	protein complex biogenesis	P	0.004853524	2.47E-05	16	119	over
GO:0016757	transferase activity, transferring glycosyl groups	F	0.007606364	4.15E-05	40	533	over
GO:0043623	cellular protein complex assembly	P	0.007606364	4.17E-05	15	111	over
GO:0005783	endoplasmic reticulum	C	0.008778652	4.98E-05	19	171	over
GO:0005887	integral to plasma membrane	C	0.011501813	6.75E-05	6	15	over
GO:0071822	protein complex subunit organization	P	0.013632918	8.27E-05	16	133	over

GO:0031226	intrinsic to plasma membrane	C	0.018746283	1.17E-04	8	35	over
GO:0035556	intracellular signal transduction	P	0.020884908	1.35E-04	25	286	over
GO:0015630	microtubule cytoskeleton	C	0.020964037	1.42E-04	16	140	over
GO:0033036	macromolecule localization	P	0.020964037	1.44E-04	40	567	over
GO:0035434	copper ion transmembrane transport	P	0.024252644	1.76E-04	5	11	over
GO:0005375	copper ion transmembrane transporter activity	F	0.024252644	1.76E-04	5	11	over
GO:0034613	cellular protein localization	P	0.030548226	2.27E-04	24	279	over
GO:0003924	GTPase activity	F	0.035015941	2.67E-04	13	104	over
GO:0070727	cellular macromolecule localization	P	0.036967856	2.89E-04	24	284	over
GO:0000139	Golgi membrane	C	0.043524332	3.49E-04	8	42	over
GO:0003779	actin binding	F	0.044599896	3.76E-04	11	80	over
GO:0045184	establishment of protein localization	P	0.044599896	3.84E-04	31	420	over
GO:0015031	protein transport	P	0.044599896	3.84E-04	31	420	over
GO:0009678	hydrogen-translocating pyrophosphatase activity	F	0.048409828	4.26E-04	4	7	over
GO:0031224	intrinsic to membrane	C	6.72E-12	2.76E-15	114	1320	over
GO:0016021	integral to membrane	C	6.72E-12	2.85E-15	110	1250	over
GO:0016020	membrane	C	1.64E-11	1.05E-14	199	3020	over
GO:0044425	membrane part	C	9.96E-11	8.45E-14	127	1634	over
GO:0008565	protein transporter activity	F	9.07E-04	9.62E-07	15	75	over
GO:0034613	cellular protein localization	P	0.002849246	3.62E-06	25	217	over
GO:0070727	cellular macromolecule localization	P	0.003043904	4.52E-06	25	220	over
GO:0035251	UDP-glucosyltransferase activity	F	0.004649592	7.89E-06	15	91	over
GO:0016760	cellulose synthase (UDP-forming) activity	F	0.008270125	1.58E-05	8	24	over
GO:0071554	cell wall organization or biogenesis	P	0.008644858	1.85E-05	23	210	over
GO:0016759	cellulose synthase activity	F	0.008644858	2.02E-05	8	25	over
GO:0006886	intracellular protein transport	P	0.01330291	3.46E-05	22	204	over
GO:0046527	glucosyltransferase activity	F	0.01330291	3.94E-05	15	106	over
GO:0005856	cytoskeleton	C	0.01330291	3.99E-05	20	176	over
GO:0044430	cytoskeletal part	C	0.01330291	4.33E-05	17	134	over
GO:0005874	microtubule	C	0.01330291	4.51E-05	13	82	over
GO:0070882	cellular cell wall organization or biogenesis	P	0.015194156	5.57E-05	17	137	over

GO:0051641	cellular localization	P	0.015194156	5.80E-05	29	326	over
GO:0005975	carbohydrate metabolic process	P	0.024480801	9.86E-05	54	806	over
GO:0004672	protein kinase activity	F	0.024679745	1.05E-04	74	1218	over
GO:0007018	microtubule-based movement	P	0.025269752	1.14E-04	12	78	over
GO:0007047	cellular cell wall organization	P	0.025269752	1.18E-04	15	118	over
GO:0045229	external encapsulating structure organization	P	0.028610906	1.40E-04	15	120	over
GO:0030244	cellulose biosynthetic process	P	0.029394554	1.52E-04	8	35	over
GO:0046907	intracellular transport	P	0.029394554	1.56E-04	24	261	over
GO:0008104	protein localization	P	0.033314441	1.84E-04	30	368	over
GO:0016773	phosphotransferase activity, alcohol group as acceptor	F	0.03425929	1.96E-04	80	1373	over
GO:0030243	cellulose metabolic process	P	0.035677418	2.12E-04	8	37	over
GO:0051649	establishment of localization in cell	P	0.045821793	2.82E-04	26	307	over

C: Cellular Component; P: Biological Process; F: Molecular Function

GO enrichment analysis was performed using Blast2go software.

Supplementary Table 14. GO enrichment analysis of highly expressed genes in ovule

GO-ID	Term	Category	FDR	P-Value	#Test	#Ref	Over/Under
GO:0019222	regulation of metabolic process	P	1.25E-05	2.55E-09	77	1515	over
GO:0005811	lipid particle	C	1.25E-05	4.87E-09	6	2	over
GO:0003677	DNA binding	F	5.34E-05	3.14E-08	103	2414	over
GO:0005634	nucleus	C	1.39E-04	1.09E-07	89	2032	over
GO:0012511	monolayer-surrounded lipid storage body	C	1.58E-04	1.54E-07	5	2	over
GO:0060255	regulation of macromolecule metabolic process	P	2.23E-04	2.62E-07	62	1253	over
GO:0032774	RNA biosynthetic process	P	2.36E-04	3.24E-07	68	1438	over
GO:0006355	regulation of transcription, DNA-dependent	P	2.55E-04	4.03E-07	57	1125	over
GO:0051252	regulation of RNA metabolic process	P	2.55E-04	4.49E-07	57	1129	over
GO:0031326	regulation of cellular biosynthetic process	P	2.96E-04	5.89E-07	58	1168	over
GO:0009889	regulation of biosynthetic process	P	2.96E-04	6.38E-07	58	1171	over
GO:0010556	regulation of macromolecule biosynthetic process	P	3.34E-04	8.49E-07	57	1153	over
GO:2000112	regulation of cellular macromolecule biosynthetic process	P	3.34E-04	8.49E-07	57	1153	over
GO:0065007	biological regulation	P	3.74E-04	1.09E-06	100	2506	over
GO:0080090	regulation of primary metabolic process	P	3.74E-04	1.10E-06	62	1310	over
GO:0031323	regulation of cellular metabolic process	P	3.95E-04	1.24E-06	62	1315	over
GO:0006351	transcription, DNA-dependent	P	3.98E-04	1.32E-06	66	1438	over
GO:0010468	regulation of gene expression	P	4.86E-04	1.78E-06	57	1182	over
GO:0051171	regulation of nitrogen compound metabolic process	P	4.86E-04	1.80E-06	58	1212	over
GO:0019219	regulation of nucleobase, nucleoside, nucleotide and nucleic acid metabolic process	P	8.18E-04	3.20E-06	57	1206	over
GO:0050789	regulation of biological process	P	0.001199459	4.93E-06	94	2397	over
GO:0045735	nutrient reservoir activity	F	0.001590129	6.84E-06	7	24	over
GO:0016602	CCAAT-binding factor complex	C	0.00821046	3.69E-05	4	5	over
GO:0043086	negative regulation of catalytic activity	P	0.008901959	4.18E-05	14	150	over
GO:0006261	DNA-dependent DNA replication	P	0.008919009	4.36E-05	6	22	over

GO:0000079	regulation of cyclin-dependent protein kinase activity	P	0.009597489	5.33E-05	3	1	over
GO:0003896	DNA primase activity	F	0.009597489	5.33E-05	3	1	over
GO:0071900	regulation of protein serine/threonine kinase activity	P	0.009597489	5.33E-05	3	1	over
GO:0044092	negative regulation of molecular function	P	0.009597489	5.44E-05	14	154	over
GO:0019901	protein kinase binding	F	0.010292779	6.04E-05	4	6	over
GO:0004857	enzyme inhibitor activity	F	0.012613196	7.65E-05	13	139	over
GO:0016070	RNA metabolic process	P	0.014231081	8.91E-05	69	1749	over
GO:0003700	sequence-specific DNA binding transcription factor activity	F	0.01983475	1.32E-04	36	741	over
GO:0001071	nucleic acid binding transcription factor activity	F	0.01983475	1.32E-04	36	741	over
GO:0019900	kinase binding	F	0.020017571	1.37E-04	4	8	over
GO:0004312	fatty acid synthase activity	F	0.02559089	1.80E-04	5	18	over
GO:0050794	regulation of cellular process	P	0.029488335	2.13E-04	79	2140	over
GO:0007049	cell cycle	P	0.030178312	2.24E-04	11	115	over
GO:0050790	regulation of catalytic activity	P	0.033757873	2.57E-04	19	299	over
GO:0043231	intracellular membrane-bounded organelle	C	0.03470739	2.72E-04	125	3780	over
GO:0003677	DNA binding	F	1.41E-04	2.98E-08	72	1512	over

C: Cellular Component; P: Biological Process; F: Molecular Function

GO enrichment analysis was performed using Blast2go software.

Supplementary Table 16. Expression variance of genes in different tissues of *G. barbadense*

Samples	Total biased genes	A_t>D_t	A_t=D_t	A_t<D_t	A_t/D_t>1.5	D_t/A_t>1.5
Ovule 0 DPA	14,671	7,374	53	7,244	3,471	3,436
Ovule 5DPA	15,269	7,549	100	7,620	3,698	3,739
Ovule 30 DPA	13,736	6,831	91	6,814	3,284	3,321
Fiber 5DPA	13,866	6,967	27	6,892	3,120	3,209
Fiber 10DPA	13,895	6,901	62	6,932	3,369	3,415
Fiber 20DPA	12,945	6,494	32	6,419	3,261	3,278
Fiber 30DPA	12,065	6,014	16	6,035	2,980	3,081
Root	13,021	6,480	155	6,386	4,164	4,075
Stem	15,073	7,591	67	7,415	3,821	3,821
Bud	15,030	7,528	69	7,433	3,995	4,157
Young leaf	13,733	6,945	74	6,754	3,715	3,666
Old leaf	13,973	6,954	89	6,930	4,020	4,022

Genes with RPKM>=1 are included in this assay. Those with absolute value of ratio (RPKM of A_t to $D_{t)}$ >1.5 are defined A_t biased and vice versa.

Supplementary Table 17. Transcription factor genes identified in A_t/D_t of *G. barbadense* genome

Family	Numbers in A_t	Average RPKM Ovule	Fiber	Numbers in D_t	Average Ovule	RPKM Fiber	Gr
AP2	42	300.03	86.70	28	422.15	140.45	32
ARF	30	519.64	298.93	32	930.07	436.98	36
ARR-B	20	78.87	46.08	24	148.94	119.26	20
B3	80	779.27	344.67	72	1,074.20	442.03	79
BBR-BPC	7	201.70	65.09	7	371.72	118.70	8
BES1	11	226.64	132.52	13	327.97	191.88	11
bHLH	210	2,042.39	971.79	196	2,762.32	1,152.82	225
bZIP	101	1,837.77	1,003.32	101	2,664.29	1,579.77	115
C2H2	156	1,221.29	855.92	157	1,930.99	1,059.42	157
C3H	68	1,064.62	832.36	71	1,953.71	1,233.25	72
CAMTA	9	187.75	117.74	9	156.89	108.52	11
CO-like	17	123.54	74.28	18	231.51	155.86	21
CPP	17	176.92	81.81	15	186.69	99.71	11
DBB	15	109.57	92.90	12	51.90	40.72	14
Dof	54	284.13	245.78	56	358.94	416.97	60
E2F/DP	12	141.48	58.86	16	159.37	50.76	10
EIL	8	278.20	258.58	8	357.52	331.02	10
ERF	217	2,335.71	1,103.18	215	2,704.12	1,395.71	225
FAR1	34	315.59	198.45	33	441.28	263.91	35
G2-like	75	766.62	335.86	72	951.03	426.92	71
GATA	39	527.98	278.60	41	890.79	410.87	46
GeBP	8	298.87	316.35	6	91.32	88.90	8
GRAS	86	734.52	479.88	82	930.47	591.38	81
GRF	15	329.02	115.48	17	545.81	151.28	18
HB-other	19	187.95	112.04	17	413.93	164.58	14
HB-PHD	1	9.57	4.33	3	52.68	23.85	4
HD-ZIP	76	1,421.94	616.47	75	2,035.10	948.45	80
HRT-like	4	24.85	23.01	3	23.34	14.82	1
HSF	34	276.36	126.16	33	414.32	151.78	39
LBD	64	397.41	140.46	63	374.48	174.99	67
LFY	1	0.14	0.06	2	0.36	0.06	1
LSD	4	102.49	66.97	5	183.47	197.21	6
MIKC	24	1,591.80	934.08	25	3,311.49	1,143.03	50
M-type	51	762.25	212.81	73	466.68	172.12	78
MYB	204	1,685.91	1,385.33	161	2,990.73	1,642.56	235
MYB_related	118	1,644.10	735.07	128	2,194.15	917.72	71
NAC	151	865.16	684.96	147	1,385.92	937.15	153
NF-X1	2	18.08	16.33	1	8.59	8.86	2
NF-YA	18	180.35	66.47	17	218.86	74.54	15

NF-YB	28	725.58	260.11	19	452.28	171.39	24
NF-YC	13	310.51	153.75	10	267.13	101.76	14
Nin-like	18	96.85	61.88	19	207.15	111.12	20
NZZ/SPL	1	0.24	0.00	1	3.47	0.12	1
RAV	8	38.57	11.77	8	80.73	35.66	9
S1Fa-like	1	68.90	115.14	3	206.53	211.87	4
SAP	4	0.03	0.00	4	0.17	0.01	2
SBP	28	319.14	194.38	26	306.97	233.28	30
SRS	13	32.33	25.14	12	35.92	17.44	13
STAT	1	5.22	2.54	1	10.89	4.92	1
TALE	34	334.75	223.20	29	320.15	175.86	44
TCP	31	255.34	253.40	36	513.88	630.43	38
Trihelix	51	833.42	486.85	51	1,138.28	654.79	51
VOZ	2	72.93	54.06	2	29.38	29.16	3
WOX	20	75.59	25.49	15	80.37	38.14	20
WRKY	110	896.36	419.20	108	1,612.38	746.59	119
YABBY	13	80.85	6.56	11	125.14	7.34	12
ZF-HD	24	342.09	116.07	25	796.78	320.67	23

Supplementary Table 18. The (+)-δ-cadinene synthase (CDN) family genes in cotton genomes

Gene ID	*G. arboreum*	*G. raimondii*	*G. hirsutum*		*G. barbadense*	
			A_t	D_t	A_t	D_t
CDN-A	3	2	1	1	1	1
CDN-B	3	2	3	-	2	3
CDN-C	6	5	2	3	3	5
CDN-D	-	1	-	1	1	1
CDN-E	2	1	1	1	3	-
Total	14	11	7	6	10	10

Supplementary Table 19. Samples used for RNA-seq

Sample	Sample No.
Germinating seeds (24 h to 120 h mixed)	Sample1
Stem (1 month post-germination)	Sample2
Young leaf (top two from 1-month-old plant)	Sample3
Old leaf (from plant at flowering stage)	Sample4
Flower and flower bud (-3 to 0 DPA)	Sample5
Leaf bud	Sample6
Ovule -3 DPA	Sample7
Ovule -2 DPA	Sample8
Ovule -1 DPA	Sample9
Ovule 0 DPA	Sample10
Ovule 1 DPA	Sample11
Ovule 2 DPA	Sample12
Ovule 3 DPA	Sample13
Ovule 5 DPA	Sample14
Ovule 10 DPA	Sample15
Ovule 20 DPA	Sample16
Ovule 30 DPA	Sample32
Ovule 35 DPA	Sample17
Ovule 45 DPA	Sample33
Fiber 5 DPA	Sample18
Fiber 10 DPA	Sample19
Fiber 20 DPA	Sample20
Fiber 30 DPA	Sample21
Root (12 h post germination)	Sample27
Root (24 h post germination)	Sample28
Root (48 h post germination)	Sample29
Root (96 h post germination)	Sample30
Root (144 h post germination)	Sample31

DPA: days post-anthesis.
